# 1000-fold enhancement in proton conductivity of a MOF using post-synthetically anchored proton transporters

**DOI:** 10.1038/srep32489

**Published:** 2016-08-31

**Authors:** Sorout Shalini, Vishal M. Dhavale, Kavalakal M. Eldho, Sreekumar Kurungot, Thallaseril G. Ajithkumar, Ramanathan Vaidhyanathan

**Affiliations:** 1 Department of chemistry, Indian Institute of Science Education and Research, Pune 411008, India; 2 Physical and Materials Chemistry Division, CSIR-National Chemical Laboratory, Pune 411008, India; 3 Central NMR Facility, CSIR-National Chemical Laboratory, Pune 411008, India; 4 Center for Energy Science, Indian Institute of Science Education and Research, Pune 411008, India

## Abstract

Pyridinol, a coordinating zwitter-ionic species serves as stoichiometrically loadable and non-leachable proton carrier. The partial replacement of the pyridinol by stronger hydrogen bonding, coordinating guest, ethylene glycol (EG), offers 1000-fold enhancement in conductivity (10^−6^ to 10^−3^ Scm^−1^) with record low activation energy (0.11 eV). Atomic modeling coupled with ^13^C-SSNMR provides insights into the potential proton conduction pathway functionalized with post-synthetically anchored dynamic proton transporting EG moieties.

In the pursuit of alternative energy, fuel cells are a key candidate. In an attempt to improve the performance of the existing fuel cells new materials are being explored as electrode or electrolyte candidates. Of the many applications that Metal Organic Frameworks (MOFs) have been investigated for, their use as proton conducting electrolyte membrane is quite recent and is being researched extensively^1^,^2^,^3^. They serve as excellent systems to compartmentalize the contribution of various modular components towards the overall proton conductivity of these materials^3^,^4^,^5^. A facile and effective method to tune proton conduction is to introduce amphoteric guests that provide a proton conducting pathway via hydrogen bond type interactions. Such strategies have enabled the realization of significant improvement in proton conduction even in many classical polymers^6^,^7^,^8^,^9^. However, their amorphous nature makes it difficult to gain structural insights and thereby limits the ability to build a structure-property relationship. On the contrary, exploiting the highly ordered porous and crystalline nature of MOFs, Shimizu and co-workers^10^ and Kitagawa and co-workers^11^ in their pioneering work have introduced extra-framework amphoteric guests to achieve moderate temperature (100–200 °C) conductivity. Most importantly, their approach coupled with the wealth of atomic level details available from the single crystal structures of these MOFs have led to superior proton conducting MOFs (PC-MOFs)^12^,^13^ formed by tailored hydrogen bonding pathways. In most cases, the hydrogen bond pathway has the ‘non-coordinated’ guest serving as an extra-framework hydrogen bond point^5^,^14^,^15^. Acidities of such guests, their position and orientation seem to play a key role in achieving high proton conductivities and low activation energies^1^,^3^,^5^. Given the vast library of guest species and easy guest loading, which is generally done by soaking the MOF in the solution of the guest molecules or exposing the activated MOF to the vapor phase of the guest, this approach certainly has versatility^10^,^11^. However, on top, if the protic groups can be made a part of the framework it would bring additional advantages^16^, particularly in terms of achieving consistent compositions and non-leachable loadings. This would favor stoichiometric loading of conducting species, which impacts the reproducible performance.

As mentioned earlier, hydrogen bonds play a key role in deciding the magnitude and charge transfer kinetics of the proton conduction in these crystalline solids. For realizing high conductivities, a major requirement is the percolation of these hydrogen bond pathways and the need for the charge transporting components to have compatible acid-base character. Post synthetic modification has become an effective approach to enhance specific properties within MOF networks^16^,^17^. A post-synthetic incorporation of flexible hydrogen bonding groups into the framework of a PC-MOF should be possible, however the conduction enhancement it can bring is not easy to predict. To the best of our knowledge, there is only one report of a post synthetic anchoring of protic species (histidine) in a MOF, which provided conductivities of the order of 10^−9^ Scm^−1^ at 146 °C and the humidity dependent conductivity was not reported^18^. However, the potential of MOF electrolytes to deliver high conductivities in the order of 10^−2^ Scm^−1^ has recently been demonstrated^13^,^19^,^20^,^21^,^22^,^23^,^24^, which has really triggered the interest in exploring different chemical/structural modifications to push the conductivities to record levels.

In this backdrop, here we present an approach wherein ‘zwitterionic’ 4-pyridinol has been used as coordinating proton source favoring stoichiometric loading and significant conductivities. Remarkably, the proton conduction of the resulting MOF has been enhanced by 1000 folds via a post synthetic loading of ethylene glycol (EG) molecules. We have isolated two isomorphous dense metal-terephthalate (m = Mg, Cd) MOFs comprising metal-coordinated pyridinol and also, a Nd-terephthalate with a very closely related structure as pure phases (Figure s1). These isostructural MOFs provide an excellent platform to understand the structure-conductivity relation in these solids controlled by the different modules within them.

## Results and Discussions

### Structure of the proton conducting metal organic frameworks (1–3)

**Figure 1:**
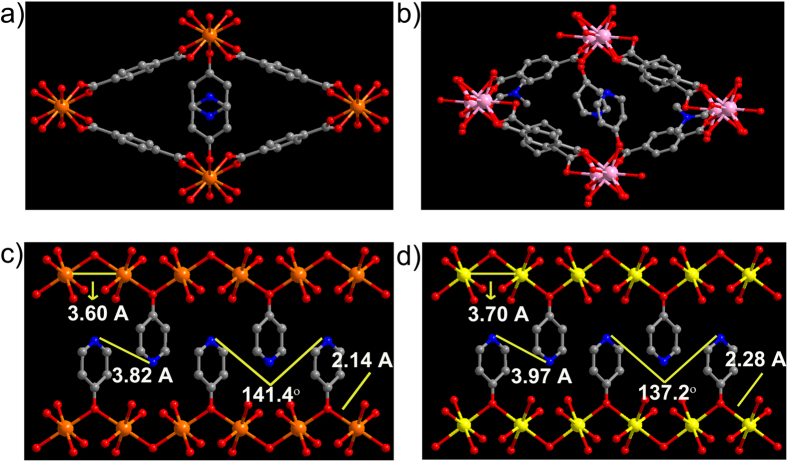
(**a**) Structure of **1** showing the rhombic shaped channel with pendant pyridinol units protruding into it. (**b**) Structure of **3** showing 1-D channel decorated with terminal pyridinol units. (**c,d**) show the inorganic chains in **1** and **2**, built from μ-2 bridging pyridinol units and their similarity can be seen from their distances and angles. N···N distances in **3** can be found in Figure s4. The terephthalate ligands connecting these chains have not been shown for clarity. Color scheme: Mg– Orange; Nd– Pink; Cd– Yellow; O– Red; C– Grey; N– Blue.

Structure of **1**, Mg(C_8_O_4_H_4_)(C_5_NOH_5_) is made up of M-O chains formed by the metal and the μ-2 bridging pyridinol (PyOH) oxygen and the carboxylate units (Fig. 1). Four such chains running along the c-axis are connected together by the terephthalate (Tp) units to form rhombic shaped 1-D channels along the c-axis (Fig. 1a). The terminally coordinated pyridinol units protrude into these channels. The pyridinol groups positioned along the c-axis are coordinated to the metal lining the top and bottom of the channel. This gives a zig-zag arrangement to the protonated pyridyl groups running along the 1D channel. Compound **2**, Cd(C_8_O_4_H_4_)(C_5_NOH_5_) is isostructural with **1**. Meanwhile **3**, Nd_2_(C_8_O_4_H_4_)_3_(C_5_NOH_5_)_2_.(DMF)_x_ has a different building unit, a Nd_2_ dimer built from two 9-coordinated Nd centers. Both have three different carboxylates- a bidentately coordinated, a μ-2 bridging monodentately chelating and a μ-2 bridging mono and bidentately chelating carboxylate. (Figure s2). In addition, both have coordination from terminal pyridinol unit and a DMF molecule. The connectivity between these dimers via the terephthalate ligands complete the 3-D structure with a narrow 1-D channel along the c-axis completely filled by pyridinol and coordinated DMF molecules (Fig. 1b). One noticeable difference is that the pyridinol is not μ-2 bridging in this case. The pyridyl ends are protonated in **1**, **2** and **3**. Also, in **1** and **2** the adjacent pyridinols are held in position via π-π interactions (π-π distance = ~3.6–3.9 Å). The bulk compositions reflecting the stoichiometric loading of pyridinol have been confirmed from TGA and CHN values (Supporting Information, Figure s3).

### Proton conductivity studies

**Figure 2:**
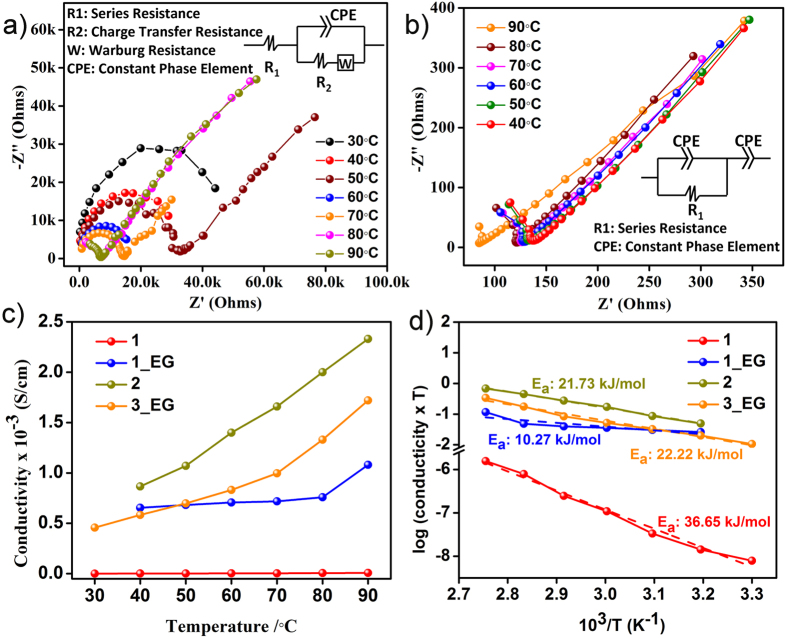
Cole-cole plots for (**a**) 1 and (**b**) 1_EG showing the variation of resistances with temperature at 90%RH. (equivalent circuits are shown as insets for both the samples) (**c**) Conductivity vs. temperature plots showing the Arrhenius behavior in all phases. (**d**) Logarithmic plot of conductivity vs. temperature.

The coordinating nature of the pyridinol positions the protonated pyridyl ends in an ordered fashion to provide a gas-tight percolating pathway for protons to move (negligible porosity, see appendix in Supporting Information). To assess the ability of the samples to act as solid electrolytes, we measured the alternating-current (ac) impedance spectrum of the pelletized samples from 30 to 90 °C at 90%RH. The conductivities for **1**, **2** and **3** were calculated by fitting the impedance spectra of the samples with a proposed equivalent circuit (Figs 2 and s5). When the conductivities were compared, it turned out that **2** made up of Cd centers showed highest conductivity (10^−3^ Scm^−1^ at 90 °C and 90% RH) among the three (**1**: Mg analogue (10^−6^ Scm^−1^ at 90 °C and 90%RH) and **3**: Nd phase (10^−5^ Scm^−1^ at 90 °C and 90%RH, Table s1).

Here, a relatively mild acid, pyridinol (pKa, = 3.2), is able to provide conductivities quite comparable to those reported in some of the sulfonic^10^ and phosphonic^15^ acid (pKa~0.6 to 2.4) based electrolytes. Higher acidities and concentration of the protic groups have been suggested as a requirement to ensure efficient proton conduction and transport^25^,^26^. For this purpose sulfonic acids and polyprotic phosphonic acids have been laced in to MOFs for gaining appreciable conductivities^10^,^15^,^16^. In comparison, we have much lesser concentration/density of protons, yet, under humid conditions, a comparable proton conduction is observed.

**Figure 3:**
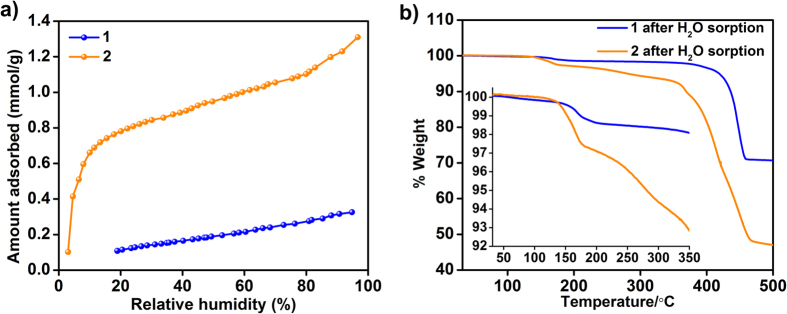
(**a**) Water-vapor adsorption isotherms of **1** and **2** at 303 K. (**b**) TGA plots of the post water-vapor sorption phases of **1** and **2** showing weight losses corresponding to water loss (120–280 °C). Inset: Zoomed-in image showing the weight losses from water.

The marked difference in the humidity dependent conductivities of **1** and **2** despite their isostructural frameworks is intriguing. Such differences can be expected to arise from the amount of water that can be accommodated within these framework structures^27^. To explore this, we have carried out water vapor adsorption on both **1** and **2**, and the isotherm data has been presented in Fig. 3.

As can be seen due to the ability of Cd centers to accommodate larger number of coordinated water compared to the coordinatively saturated octahedral Mg in **1**, the Cd compound, **2**, shows a water uptake ~8 times higher. Also, when the post water sorption samples were analyzed under the TGA (Note: Significant number of the water molecules have been desorbed during the desorption cycle of the experiment), it could be seen that there was clear weight loss corresponding to water for both **1** and **2**, which were not present in the as-synthesized phases. When these hydrated forms of **1** and **2** are compared it can be seen that the weight loss due to water is observed in the temperature range of 120 to 200 °C in **1**, while **2** shows a two step weight loss in the temperature range of 130 to 280 °C. These water losses at higher temperatures are likely to be associated with tightly bound water and some of them could be coordinated to the metal site. Most importantly, the compound **2**, clearly seems to accommodate significantly larger amounts of water compared to **1** (TGA weight loss: 2% for **1** vs 8% for **2,** inset Fig. 3).The compounds were stable to the water sorption experiments as observed from PXRD.

The modular structure of the MOFs permits ligand substitution via post synthetic modification, a feature that has been well exploited in enhancing the proton conductivities in MOFs by introducing transport-mediating salts^28^, acid impregnation^16^,^19^,^29^, ion substitution^8^,^30^,^31^, isomorphous ligand replacement^32^ etc. A closer look at the structure reveals that in **1**, **2** and **3** the spatial separation of the protonated pyridinol units is quite large (3.82 Å in **1**, 3.97 Å in **2** and 5.52 Å in **3**, Fig. 1). Threading a more flexible and strongly hydrogen bonding guest along the conduction path lined by the pyridinols could assist in transferring these far-spaced protons. This necessitates identifying a protic guest with right hardness capable of binding with the metal centers of interest, preferentially over pyridinol and sufficient flexibility to facilitate proton shuttling via increased hydrogen bonds. And, most importantly it has to be neutral to be able to remove the zwitter-ionic pyridinols. We realized Ethylene Glycol (EG) satisfies all these requirements.

To load EG, we stirred **1**, Mg_4_(Tp)_4_(PyOH)_4_, in ethylene glycol at 120 °C. This procedure gets 1/4^th^ (confirmed via TGA and ^13^C-MASNMR) of the pyridinol replaced by EG giving Mg_4_(Tp)_4_(PyOH)_3_(EG). From the inherent high conductivities of **2**, it can be expected that introducing EG into it could produce very high conductivities, however, all our attempts resulted in framework collapse. **3**, however, was stable to pyridinol loss. Also, the presence of exchangeable coordinated DMF provides an additional site for EG binding. DMF was removed by heating at 100 °C under vacuum for 2 hrs. This sample was maintained under vacuum, and EG was syringed in and allowed to stand for 12 hrs at 120 °C. EG treated samples were thoroughly washed with copious amount of solvents. The glycol loaded phases, **1_EG** and **3_EG**, showed conductivities of 1.08 × 10^−3^ and 1.72 × 10^−3^ Scm^−1^, respectively at 90 °C and 90% RH. 1000-fold enhancement in proton conductivity with this simple design modification is quite remarkable.

### Structural simulation studies

**Figure 4:**
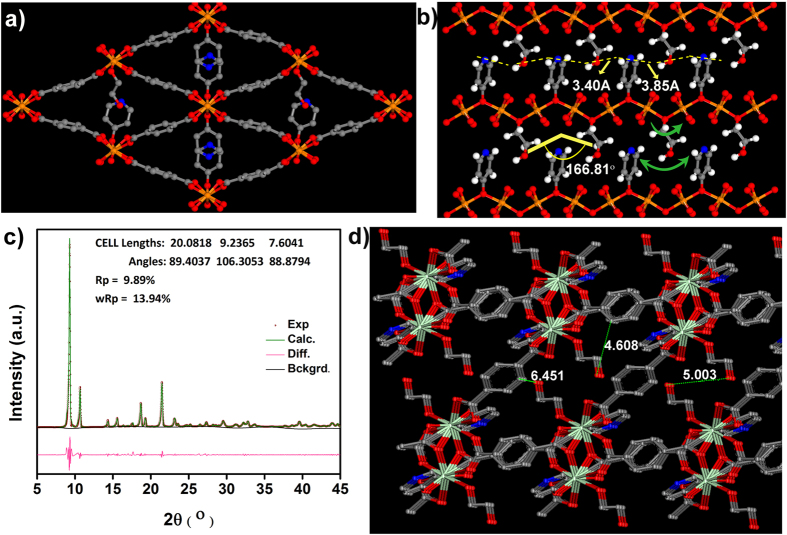
(**a**) Simulated structure of 25% EG loaded phase of 1 i.e. 1_EG, showing the pendant EG and pyridinol lining the top and bottom of the 1-D channels (hydrogens not shown for clarity). (**b**) An a-axis view showing the pendant EG and pyridinol lining the top and bottom of the 1-D channels (Dotted yellow lines: potential H-bond pathway; Green arrows: Rotational and pendulum like motion that can be possible with the EG assisting proton transfer along this pathway). (**c**) Pawley refinement carried out on **1_EG**. (**d**) Simulated structure of **3_EG**, with the DMF sites replaced by EG molecules and the energy/geometry was minimized using DFT routine. The optimized geometry shown above indicates the presence of larger spaces in this, wherein the EG resides and could have sufficient dynamic character to facilitate hydrogen bonds between protic pyridinols and can accommodate more water molecules under humid conditions. This could explain the higher conductivities of **3** over **1**. Color code: Sea-green- Nd; Red- O; Grey- C; Blue- N.

In recent times atomic simulation has been used very effectively in solving and manipulating periodic structures, particularly, in framework solids such as MOF and COF^33^,^34^,^35^,^36^. To gain further insights on the role of EG, we carried out structural simulations using Materials Studio (Accelrys). A triclinic model was built wherein 1/4^th^ of the pyridinol sites were replaced by EG molecules (consistent with TGA and SSNMR). In the case of Mg, we made one of the oxygens of the EG into a μ-2 bridging and the terminal end was left dangling into the pore, which gets sandwiched between the pyridinol units along the c-axis (Fig. 4a). Now a geometry optimization was carried out on this configuration using a tight binding DFT (DFT-TB) algorithm with keeping the unit cell fixed. This model yielded a relative energy ~50% lower than the parent phase. This is well supported by the noticeable increase in the number of weak hydrogen bonds in the EG loaded configuration as determined from a PLATON analysis (supporting info). A potential hydrogen bond pathway lined with protonated pyridinols and the EG moieties has been shown in Fig. 4b. The pendant type arrangement of this ethylene glycol group owing to its freedom of rotation and bending could allow the EG to act like a pendulum shuttling the protons across the pyridinol units. We believe, this cooperativity between the EG and the pyridinol is responsible for the 1000-fold increase in proton conductivity (10^−6^ to 10^−3^ Scm^−1^) under the humid conditions employed and seems to generate a strongly knit percolating conduction pathway favoring Grotthuss transport with extremely low activation energy (0.11 eV)^29^,^37^. In fact, this represents the minimal energy required for breaking hydrogen bonds, which would mean the pyridinol-EG combination provides almost optimal strength hydrogen bonds facilitating transport of mildly acidic protons^37^. For the Nd case, the EG were made to replace the coordination site occupied by the DMF molecules, and its geometry was optimized (Fig. 4). However, when the resultant configuration was compared to the as-synthesized Nd phase, the EG containing phase had considerable number of hydrogen bonds and a lower relative energy. We could corroborate this triclinic model of **1_EG** and **3_EG** further by carrying out both Pawley refinements and Lebail fits and the Fobs could be extracted (Figs 4, s10 and s11). The fits obtained reflected a good match between the experimental PXRD of the EG loaded phases with the proposed models.

### Solid State ^13^C-NMR studies

**Figure 5:**
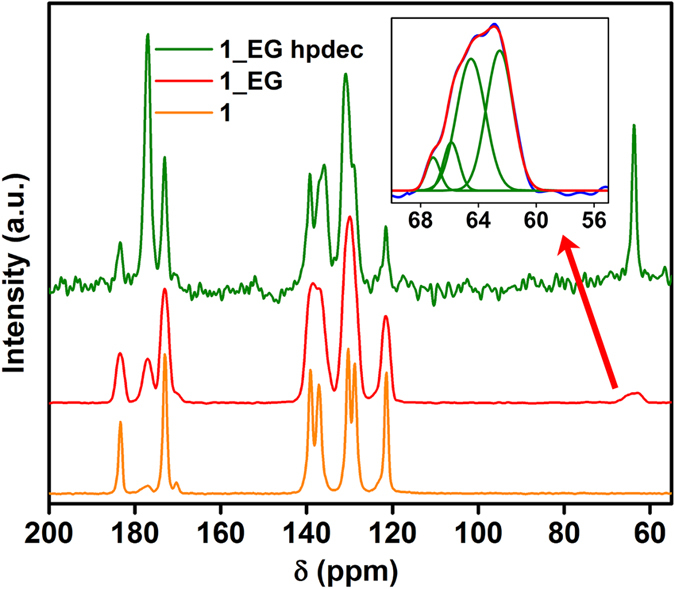
Comparison of the ^13^C-SSNMR of **1** and **1_EG,** showing the presence of multiple coordination modes for EG.

Solid state NMR has been used very effectively in understanding the role of functional groups in many proton conducting MOFs^38^,^39^. Interestingly, from solid state NMR a monodentate coordination of EG was observed for both **1_EG** and **3_EG** (Figs 5 and s12) with them being terminally bonded to the metal centers (Mg and Nd). This would leave the non-coordinated R-OH groups dangling into the pore. Further, to obtain some insights into the mobility associated with the framework components, particularly the EG and PyOH, the SSNMR patterns of **1** and **1_EG** have been compared (Fig. 5).

Signals corresponding to EG can be observed in the range of 55–70 ppm. There is a prominent peak at 64 ppm in the case of **1_EG** which is not seen in **1**. Also, the peak corresponding to the EG appears to be a quartet. A simple peak fit suggests the presence of four peaks (chemical shifts: 62, 64, 65, 67ppm; χ^2^ = 0.99561). This indicates the presence of more than one coordination modes for the EG (scheme s1). To further support the model of a singly coordinated mobile EG, we prepared a model compound, MgTpEG, wherein the EG are monodentately coordinated to two different Mg centers via both its -OH groups. Noticeably, the profile for the EG peak in this case had a singlet, as compared to the multiplets observed for the **1_EG** (Fig. s13). The ^13^C CPMAS NMR experiments on this model compound suggested the lack of any mobility associated with the EG groups. Thus, it can be concluded that there is a high probability of a singly bound EG in **1_EG**. When the spectra from ^13^C CPMAS NMR and ^13^C MAS NMR with high power decoupling (hpdec) are compared, it appears that these EGs are very mobile. A more detailed explanation of the SSNMR results can be found in the supporting information.

### Post-impedance stability of the MOFs

**Figure 6:**
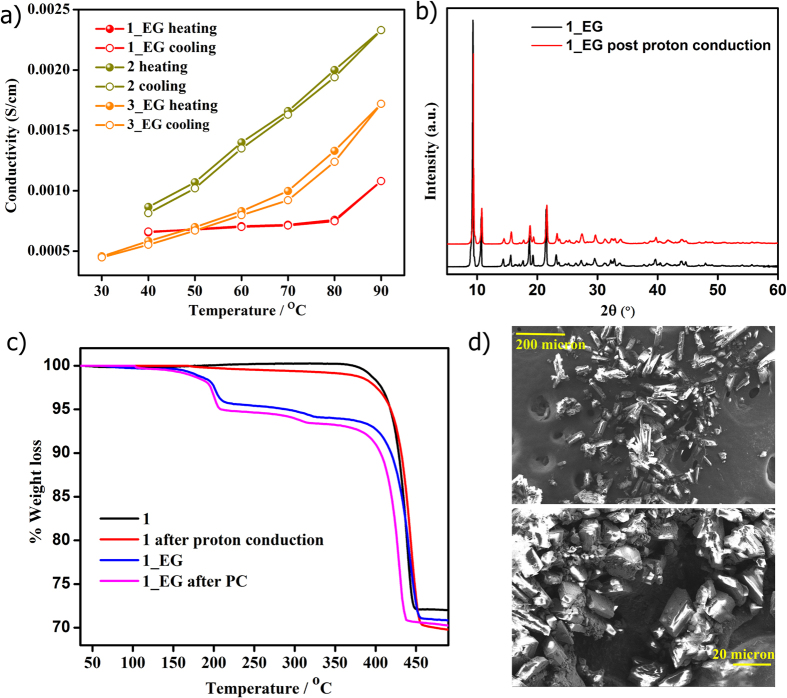
(**a**) The stability of the conductivity of **1**–**3** observed from heating and cooling cycles. Structural integrity of the highest conducting phases, **1** and **1_EG**, under the humid and high temperature conditions of the proton conduction measurements evidenced from (**b**) PXRD comparisons (**c**) TGA comparisons and (**d**) FE-SEM studies.

To verify if there was any possible guest leaching under the operational conditions, the conductivities for both heating-cooling cycles were compared, which indicated no change (Figs 6a, s14 and s15). Furthermore, characterizations using PXRD, TGA, CHN and Field Emission SEM carried out on the post-impedance measurement samples confirmed the lack of any guest leaching (Figs 6, s16–s20 and Table s2). If there is any contribution to these high conductivities in the glycol loaded phases owing to partial decomposition of the MOF phases, it would be expected that similar decomposition should have resulted in the as-made phases again giving rise to high conductivities. However, **1** clearly has significantly lower conductivities compared to the **1_EG** even under 90% rH and 90 °C, which strongly suggests lack of any such decomposition. Even the recently reported sodium and cesium sulfonate and magnesium phosphonate MOFs do not show any such decomposition under similar conditions^10^,^20^,^40^.

## Conclusion

In conclusion, this is a proof-of-concept, wherein anchoring zwitter ionic guests into MOF frameworks has been shown to be an effective strategy to achieve stoichiometric loading and a route to minimize guest leaching, which are key to obtaining consistent proton conduction. Importantly, a post-synthetic exchange of coordinating pyridinol units with another neutral and dynamic hydrogen bonding species, ethylene glycol, results in a better hydrogen-bond mediated conduction pathway with drastically enhanced conductivities and with record lowest activation energy. This strategy could be extended quite readily across several other metal-organic systems.

## Methods

**1** was synthesized hydrothermally using the approximate molar ratios Mg(NO_3_)_2_•6H_2_O, terephthalic acid and 4-hydroxypyridine (1:1:2). 0.1 g Magnesium nitrate was dissolved in 5 ml DMA. To this solution was added 0.08 g 4-hydroxypyridine. The mixture was stirred for 15 minutes at RT. To this 0.065 g terephthalic acid was added. The contents were sealed in an autoclave and heated at 140 °C for 48 hours. It was slowly cooled down to room temperature. Product containing rod-shaped crystals was collected by filtration using methanol and acetone. (Elemental analysis, observed/calculated: C – 54.80/55.07; H - 3.076/3.19; N–4.82/4.94)

**1_EG** was prepared by stirring 0.3 g of **1** in 5 ml EG at 120 °C for 12 hours. Following this, it was filtered and washed with methanol and acetone. (Elemental analysis, observed/calculated: C – 52.58/53.45; H - 3.84/3.38; N – 3.77/3.8)

**2** was synthesized hydrothermally using the approximate molar ratios Cd(NO_3_)_2_•4H_2_O, terephthalic acid and 4-hydroxypyridine (1:1:2). 0.1 g cadmium nitrate was dissolved in 5 ml DMF. To this solution was added 0.06 g 4-hydroxypyridine. The mixture was stirred for 15 minutes at RT. To this 0.0538 g terephthalic acid was added. The contents were sealed in an autoclave and heated at 100 °C for 24 hours. It was slowly cooled down to room temperature. Product containing rod-shaped crystals was collected by filtration using methanol and acetone. (Elemental analysis, observed/calculated: C – 42.65/42.01; H - 2.311/2.44; N – 3.73/3.76)

**3** was synthesized hydrothermally using the approximate molar ratios Nd_2_(CO_3_)_3_•xH_2_O, HNO_3_, terephthalic acid and 4-hydroxypyridine (1:3:1.5:3). 60 μl nitric acid was added to 0.1 g Neodymium carbonate and 5 ml DMF. To this solution was added 0.12 g 4-hydroxypyridine. The mixture was stirred for 15 minutes at RT. To this 0.106 g terephthalic acid was added. The contents were sealed in an autoclave and heated at 100 °C for 24 hours. It was slowly cooled down to room temperature. Product containing very thin rod-shaped crystals was collected by filtration using methanol and acetone. (Elemental analysis, observed/calculated: C – 42.18/42.56; H - 2.144/2.80; N – 3.99/4.02)

**3_EG** was prepared by heating **3** at 100 °C for 2 hours and then stirring it in EG for 12 hours at 120 °C. (Elemental analysis, observed/calculated: C – 41.82/42.02; H - 2.191/2.35; N – 2.69/2.72).

The bulk products were phase pure. The purity was checked using powder X-Ray diffraction experiments.

## Additional Information

**How to cite this article**: Shalini, S. *et al*. 1000-fold enhancement in proton conductivity of a MOF using post-synthetically anchored proton transporters. *Sci. Rep.* **6**, 32489; doi: 10.1038/srep32489 (2016).

## Supplementary Material

Supplementary Information
