# Supplementary material for: 1000-fold enhancement in proton conductivity of a MOF using post-synthetically anchored proton transporters
Source: Sci Rep. 2016 Aug 31;6:32489. doi: 10.1038/srep32489 (PMC5006155; doi:10.1038/srep32489)
Supplement: Supplementary Information [file srep32489-s1.pdf]

Supporting Info for

1000-fold enhancement in proton conductivity of a MOF using post-synthetically anchored proton transporters

Sorout Shalini,[a] Vishal M. Dhavale,[b] Kavalakal M. Eldho,[c] Sreekumar Kurungot,[b] Thallaseril G. Ajithkumar[c] and Ramanathan Vaidhyanathan[a,d*]

a. Department of Chemistry, Indian Institute of Science Education and Research, Dr. Homi Bhabha Road, Pashan, Pune, 411008, Maharashtra, India

b. Physical and Materials Chemistry Division, CSIR-National Chemical Laboratory, Dr. Homi Bhabha Road, Pashan, Pune, 411008, Maharashtra, India.

c. Central NMR Facility, CSIR-National Chemical Laboratory, Dr. Homi Bhabha Road, Pashan, Pune, 411008, Maharashtra, India.

d Center for Energy Science, Indian Institute of Science Education and Research, Pune 411008, India.

Analytical characterizations: TGA, Powder diffraction, IR and Solid State NMR

Powder X-ray diffraction:

Powder XRDs were carried out using a Rigaku Miniflex-600 instrument and processed using PDXL2 software.

IR spectroscopy:

IR spectra were obtained using a Nicolet ID5 attenuated total reflectance IR spectrometer. The KBr pellets were used.

Solid State NMR spectroscopy:

Solid state NMR was recorded using an 800MHz Bruker NMR instrument.

Thermogravimetric Analysis:

Thermogravimetry was carried out on NETSZCH TGA-DSC system. The TGAs were done under N_2 gas flow (20ml/min) (purge + protective) and samples were heated from RT to 550°C at 2K/min.

- **1**: $Mg(C_8O_4H_4)(C_5NOH_5)$

A weight loss of 33.50 % between 350 to 450°C corresponds to the loss of coordinated pyridinol.

- **1_EG**: $Mg_4(C_8O_4H_4)_4(C_5NOH_5)_3.(C_2H_6O_2)$

A weight loss of 5.72% around 180°C corresponds to the loss of coordinated EG. This is in agreement with the 25 % replacement of pyridinol. The second weight loss (~24.25%) corresponds to pyridinol.

- **2**: $Cd(C_8O_4H_4)(C_5NOH_5)$

A weight loss of 25.59 % from 350 to 380°C corresponds to loss of pyridinol. As inferred from TGA, *material is not stable after pyridinol loss.*

- **3**: $Nd_2(C_8O_4H_4)_3(C_5NOH_5)_2.(DMF)_x$

A weight loss of ~ 16 % at 200°C corresponds to the pyridinol loss.

Impedance measurements:

The Bio-Logic (VMP-3) model was used to carry out the impedance measurement. The impedance spectra were recorded by applying range of frequency from 1 MHz to 100 mHz with an amplitude of 10 mV. Moreover, ESPEC chamber was used to maintain the temperature and humidity while measurement.

Single crystal structure determination:

Single-crystal data was collected on a Bruker SMART APEX four-circle diffractometer equipped with a CMOS photon 100 detector (Bruker Systems Inc.) and with a Cu Kα radiation (1.5418Å). The incident X-ray beam was focused and monochromated using Micro focus (IμS). Crystal of **1, 2 and 3** were mounted on nylon Cryo loops with Paratone-N oil. Data was collected at 173(2) K for **1** and **3**, and at 298K for **2**.

Data was integrated using Bruker SAINT Software and was corrected for absorption using SADABS. Structure was solved by Intrinsic Phasing module of the Direct methods and refined using the SHELXTL 2014 software suite. All non-hydrogen atoms were located from iterative examination of difference F-maps following which the structure was refined using least-squares method. Hydrogen atoms were placed geometrically riding mode (CIF file: CCDC 1041840-1041842).

Field Emission SEM:

Ultra Plus Field Emission Scanning Electron Microscope with integral charge compensator and embedded EsB and AsB detectors. Oxford X-max instruments $80mm^2$. (Carl Zeiss NTS, Gmbh), Imagin conditions: 2kV, WD=2mm, 200kX, Inlens detector.

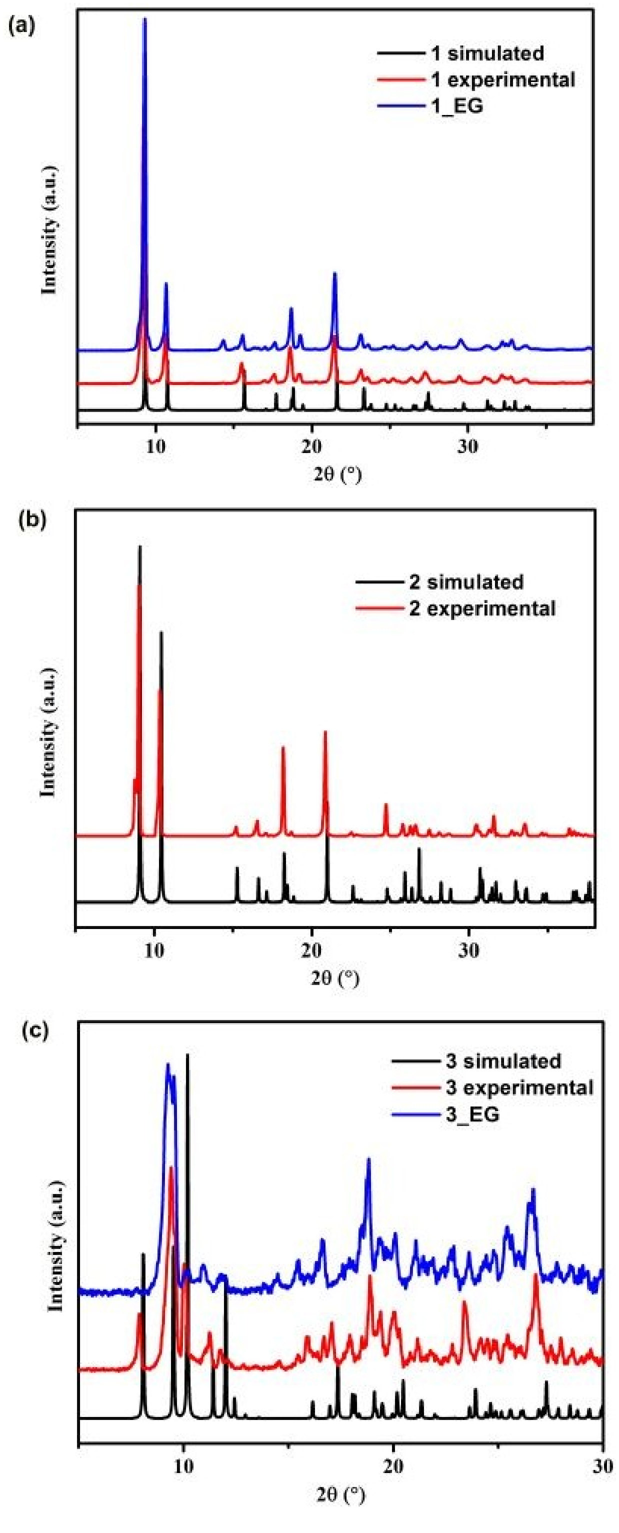

Figure s1: (a) PXRD comparison for **1** (simulated: black, as made: red) and **1_EG** (blue); (b) PXRD comparison for **2**; PXRD comparison for **3** (simulated: black, as made: red) and **3_EG** (blue). The relatively broader peaks for **3** are most likely due to fluorescence from the sample and such broad peaks are known in lanthanide MOFs. Repeated synthesis produced very similar samples with the similar PXRD profiles.

a)

b)

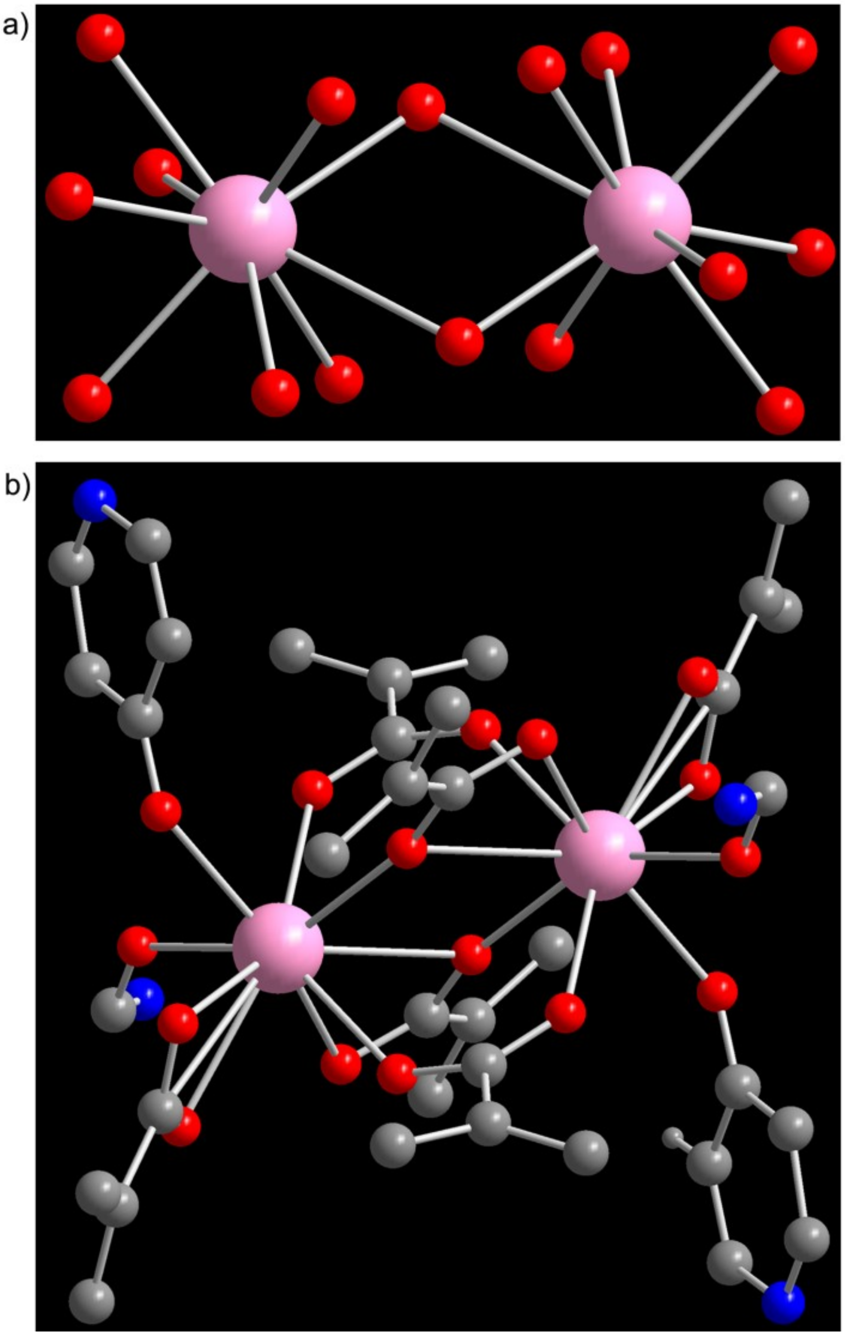

Figure s2. Coordination environment of Nd in **3** showing the Nd_2 dimers. The coordination around the dimer shows the different binding modes of the terephthalate units and also the presence of monodentately linking pyridinols and terminally coordinated DMF molecules. Color code: Pink- Nd, red- O, blue- N, Grey- C.

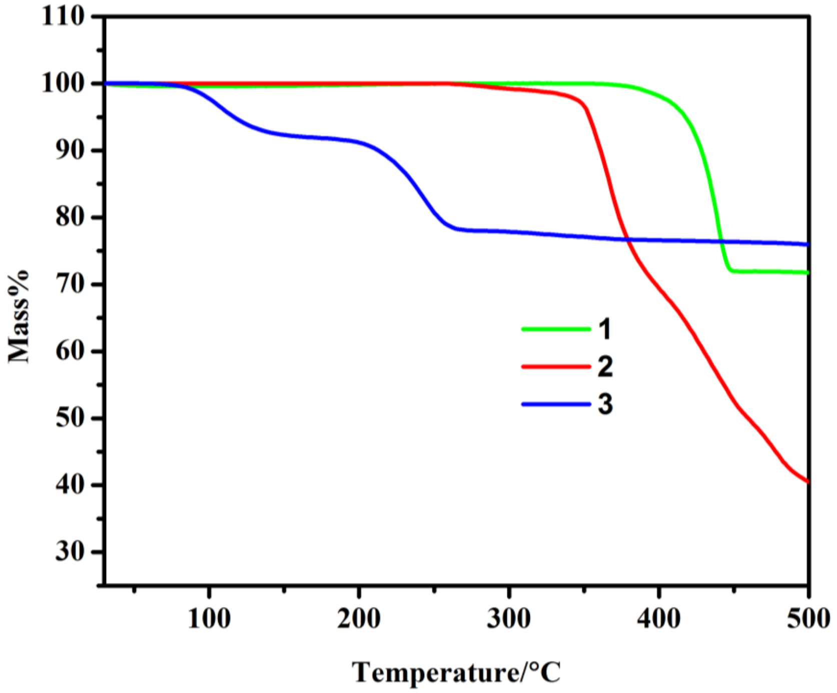

Figure s3. TGA Comparison of **1** (green), **2** (red) and **3** (blue).

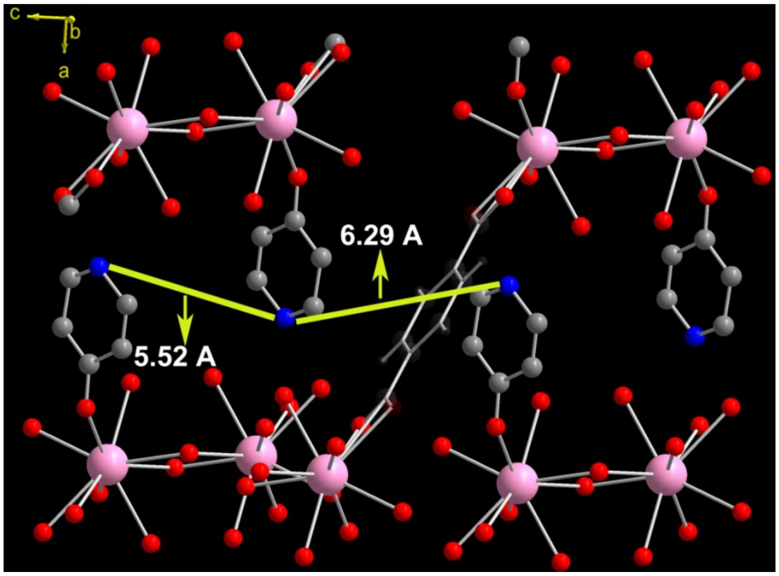

Figure s4. A column of dimers in **3**. Note the presence of pyridinols alternating up and down along the column. The dimers are connected by terephthalates to form the rhombic channels, which have not been shown for clarity. The separation between pyridinols are larger than in **1**, however this opens up a lot of space for water molecules to come in as hydrogen bonding guest under the humid conditions. This could account for its higher proton conductivity than **1**. Color code: Pink- Nd, blue- N, red- O, grey-C.

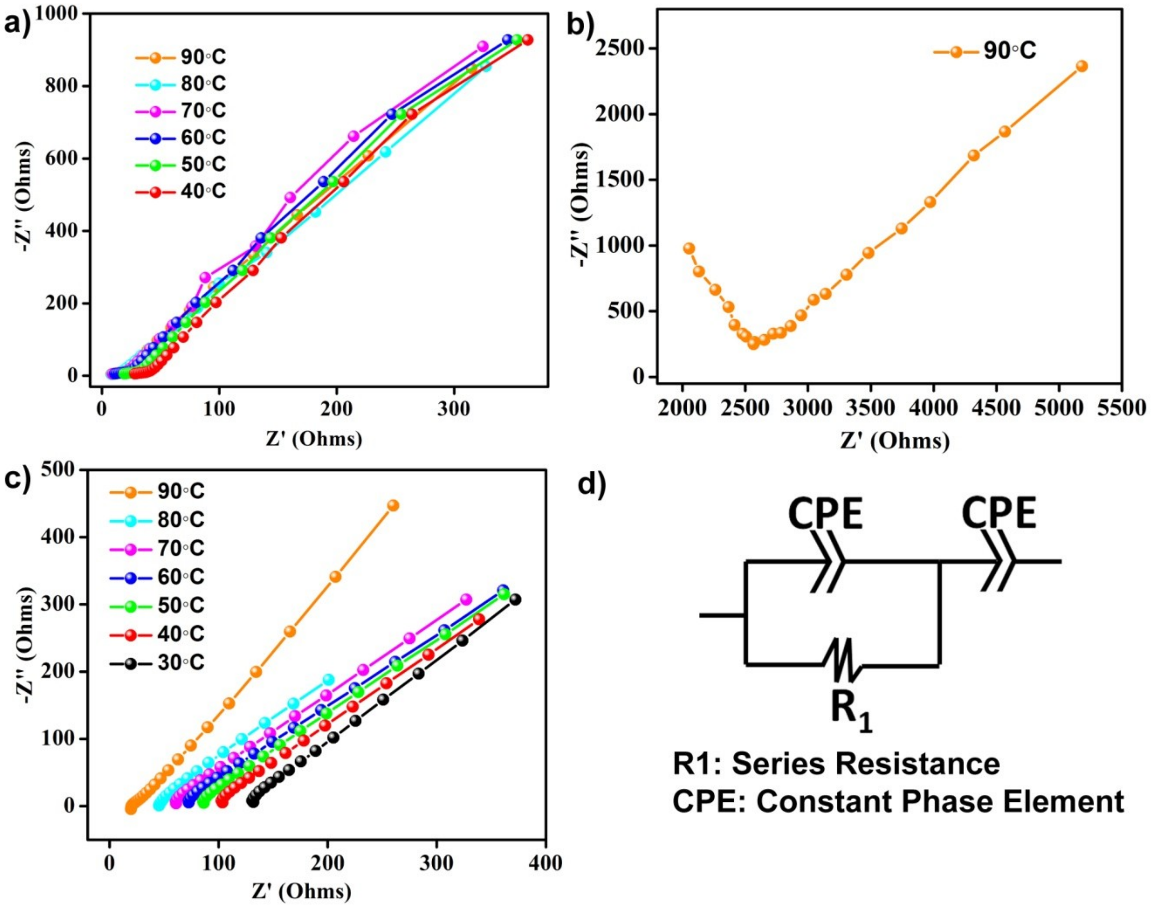

Figure s5. a) Variable Temperature Impedance Spectra for **2** at 90% RH. b) Impedance spectrum for **3** at 90% RH. c) Variable Temperature Impedance Spectra for **3_EG** at 90%RH and d) shows the equivalent circuit used to calculate resistance for **2**, **3** and **3_EG.**

Table s1. Conductivity data for **1**, **1_EG**, **2**, **3** and **3_EG.**

**Compound**	**Activation energy (eV)**	**Conductivity@ 90°C/90%RH (S/cm)**
**1**	0.38	$8.30\ x10^{-06}$
**1_EG** (heating)	0.11	$1.08\ x10^{-03}$
**1_EG** (cooling)	0.11	$1.08\ x10^{-03}$
**2** (heating)	0.22	$2.33\ x10^{-03}$
**2** (cooling)	0.23	$2.33\ x10^{-03}$
**3**	N/A	$4.04\ x10^{-05}$
**3_EG** (heating)	0.23	$1.72\ x10^{-03}$
**3_EG** (cooling)	0.21	$1.72\ x10^{-03}$

*The low activation energies in all cases suggest Grotthuss type conduction. Activation energies calculated using $\sigma = (\sigma 0/T)\ exp(-Ea/\ [R\ or\ (KB)^*\ T])$.

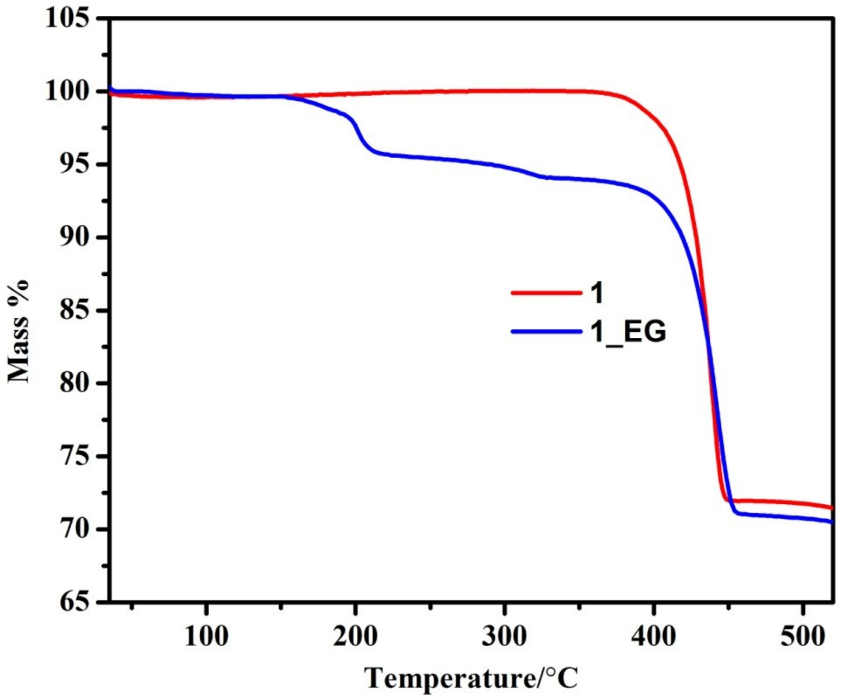

Figure s6. (a) TGA comparison of **1** and **1_EG**. Weight loss corresponding to loss of μ-2 bridging pyridinol happens in the temperature range of 320 to 450ºC and from VTPXRD it can be seen that the crystallinity is maintained.

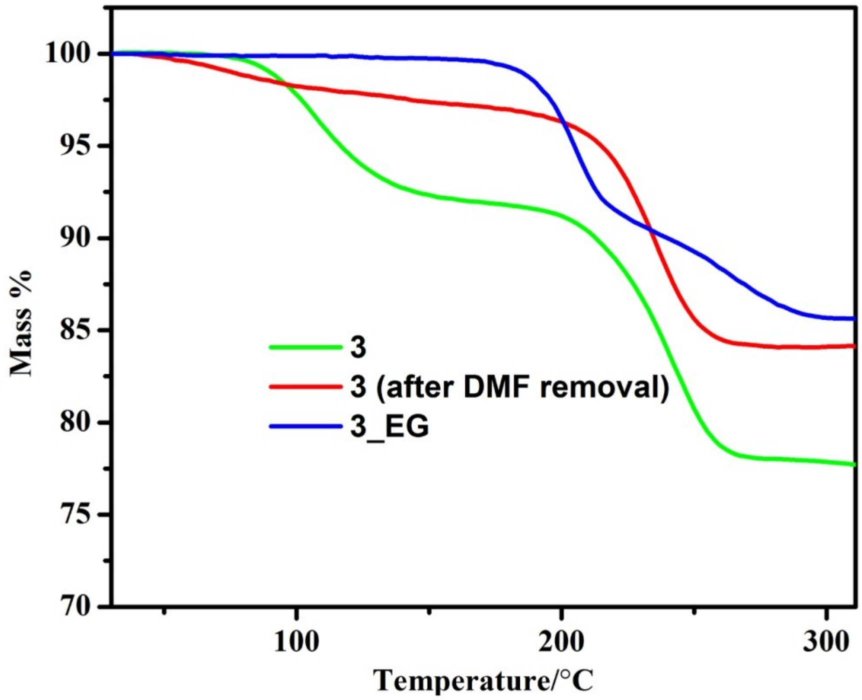

Figure s7. TGA comparison for **3**, **3** after DMF removal and **3_EG**.

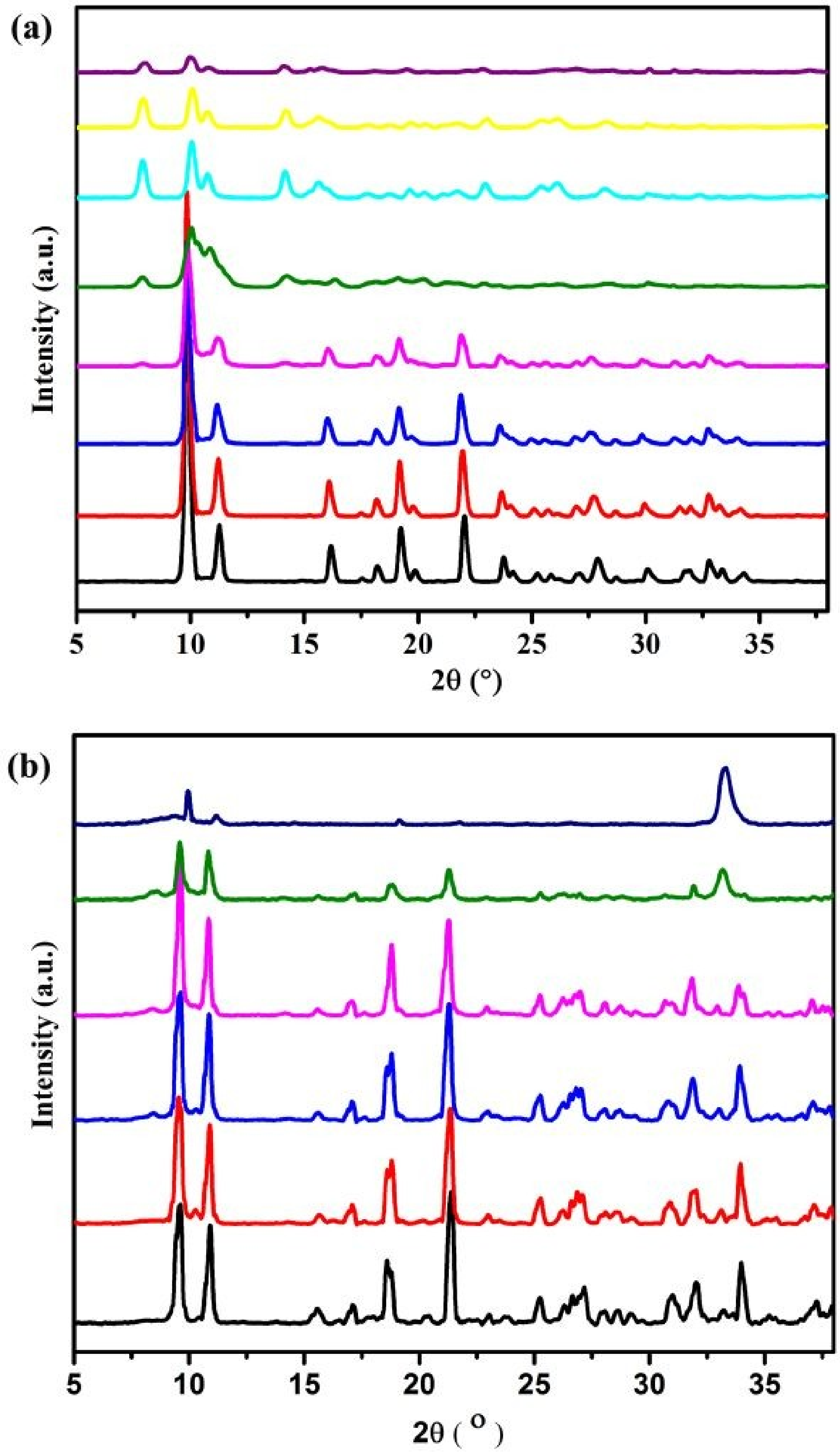

Figure s8. Variable temperature PXRD for (a) **1**. Note the change of phase starting at 450°C and a complete new phase is obtained by 550°C. This phase has been indexed (Figure s8). Black: 30, Red: 200, Blue: 380, Pink: 400, Olive: 450, Cyan: 550, Yellow: 600, Purple: 30; (b) **2**. Black: 30, Red: 150, Blue: 250, Pink: 350, Olive: 400, Purple: 450°C.

Discussion on the results from VT-PXRD experiments of 1:

The loss of pyridinol from **1** at 450°C would result in Mg with a highly unfavorable four-coordinated geometry inducing a severe structural strain. And one can expect the structure to rearrange. Accordingly, with the loss of pyridinol at 450°C, a phase change sets in and by 550°C a beta-phase crystallizes (Fig. s6). This essentially conveys that the Mg-terephthalate exists as a crystalline phase even upon the removal of the pyridinol. In order to verify if the phase formed by the complete loss of pyridinol corresponds to any of the known Mg terephtahlates, we indexed the beta phase and carried out a Pawley fit (Accelrys). This resulted in a triclinic cell for this high temperature beta phase (a= 12.006Å; b= 10.431Å; c= 10.699Å, α= 69.285°; β= 111.284°; γ= 99.029°), which corresponds to a new Mg terephthalate phase (Fig. s8).

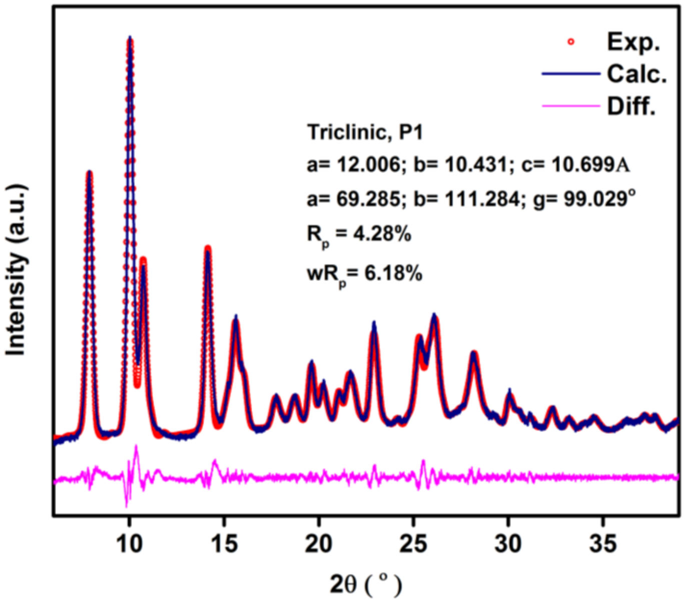

Figure s9. Pawley refinement of the new beta phase formed by the transformation of the **1** at 550°C.

Discussion on higher relative conductivity in 2 and 3: An alternate explanation to the higher conductivity observed for the as-made form of the Cd phase compared to the Mg and Nd analogues could lie in their coordination tendencies. In both **1** and **2**, the metal centers are octahedrally coordinated. While this represents a saturated coordination for Mg, Cd can carry up to seven coordinations, offering one extra site for water to coordinate under humid conditions employed during the impedance measurements. And it is well known that coordinated water itself can act as an amphoteric center to not only to hydrogen bond but also to transfer proton across a channel. Again, Nd in **3** can also accommodate more coordinated water molecules, but the relatively little difference in conductivity between **3** and **1** suggests that this effect contributes much lesser to the overall conductivity compared to the acidity differences in pyridinol arising from the hardness of the metal centers.

Hydrogen bonds analyses using PLATON (WinGX) S64_Mg_asmade, 1.
Analysis of Potential Hydrogen Bonds and Schemes with d(D...A) < R(D)+R(A)+0.50, d(H...A) < R(H)+R(A)-0.12 Ang., D-H...A > 100.0 Deg
==
Note: - ARU codes in [] are with reference to the Coordinates printed above (Possibly transformed, when MOVE .NE. 1.555)
==

Nr	Typ	Res	Donor --- H....Acceptor	[ARU]	D - H	H...A	D...A	D - H...A	A..H..A*	A'..H..A"	Sum(XY,YZ)	Sum(XZ)
1		1	C(7) --H(11) ..O(6)	[1556.01]	1.14	2.43	3.1047	116				

Translation of ARU-code to Equivalent Position Code. [1556] = x,y,1+z.

S64_Mg_EG, 1_EG
Analysis of Potential Hydrogen Bonds and Schemes with d(D...A) < R(D)+R(A)+0.50, d(H...A) < R(H)+R(A)-0.12 Ang., D-H...A > 100.0 Deg
==
Note: - ARU codes in [] are with reference to the Coordinates printed above (Possibly transformed, when MOVE .NE. 1.555)
==

Nr	Typ	Res	Donor --- H....Acceptor	[ARU]	D - H	H...A	D...A	D - H...A	A..H..A*	A'..H..A"	Sum(XY,YZ)	Sum(XZ)
1	Intra	1	C(7) --H(80) ..O(6)	[1554.01]	1.14	2.43	3.1047	116				
2	Intra	1	C(21) --H(86) ..O(20)	[ ]	1.14	2.43	3.1047	116				
3	Intra	1	C(35) --H(92) ..O(34)	[1556.01]	1.14	2.43	3.1047	116				
4	Intra	1	C(46) --H(97) ..O(71)	[1565.01]	1.14	2.47	3.1032	113				
5	Intra	1	C(49) --H(98) ..O(48)	[ ]	1.14	2.43	3.1047	116				
6	Intra	1	C(70) --H(104) ..O(27)	[1545.01]	1.14	2.59	3.0633	104				
7	Intra	1	C(70) --H(104) ..N(75)	[1544.01]	1.14	2.25	3.3540	163				

Translation of ARU-code to Equivalent Position Code. [1545] = x,-1+y,z; [1554] = x,y,-1+z; [1556] = x,y,1+z; [1565] = x,1+y,z; [1544] = x,-1+y,-1+z.

Nd_asmade, 3
Analysis of Potential Hydrogen Bonds and Schemes with d(D...A) < R(D)+R(A)+0.50, d(H...A) < R(H)+R(A)-0.12 Ang., D-H...A > 100.0 Deg
==
Note: - ARU codes in [] are with reference to the Coordinates printed above (Possibly transformed, when MOVE .NE. 1.555)
==

Nr	Typ	Res	Donor --- H....Acceptor	[ARU]	D - H	H...A	D...A	D - H...A	A..H..A*	A'..H..A"	Sum(XY,YZ)	Sum(XZ)
1		1	C(103)--H(103) ..O(3)	[1655.01]	0.93	2.58	3.1968	124				
2	Intra	1	C(11) --H(206) ..O(1)	[ ]	0.93	2.52	3.4323	166				

Translation of ARU-code to Equivalent Position Code. [1655] = 1+x,y,z

---

Nd_EG, 3_EG

Analysis of Potential Hydrogen Bonds and Schemes with d(D...A) < R(D)+R(A)+0.50, d(H...A) < R(H)+R(A)-0.12 Ang., D-H...A > 100.0 Deg

Note: - ARU codes in [] are with reference to the Coordinates printed above (Possibly transformed, when MOVE .NE. 1.555)

Nr	Typ	Res	Donor --- H....Acceptor	[ARU]	D - H	H...A	D...A	D - H...A	A..H..A*	A'..H..A"	Sum(XY,YZ)	Sum(XZ)
1	Intra	1	C(11) --H(12) ..O(31)	[ ]	1.07	2.57	2.9941	103				
2	Intra	1	C(15) --H(16) ..O(37)	[2666.01]	1.07	2.37	3.1729	130				
3	Intra	1	C(20) --H(21) ..O(34)	[2666.01]	1.07	2.31	3.2943	153				

Translation of ARU-code to Equivalent Position Code. [2666.] = 1-x,1-y,1-z.

For C--H...Acceptor Interactions See: Th. Steiner, Cryst. Rev, (1996), 6, 1.
H-Bond classification [G.A.Jeffrey, H.Maluszynska & J.Mitra., Int.J.Biol.Macromol.(1985),7,336.
2-Centre (linear) D-H...X most prob. angle 160 ° - also: G.A.Jeffrey & W.Saenger, Hydrogen Bonding in Biological Structures; 3-Centre (bifurcated) SUM of 3 angl. about H = 360°, Springer-Verlag, Berlin, 1991, pp 20. 4-Centre (trifurcated).

PXRD - Analysis: Pawley and Le Bail refinements

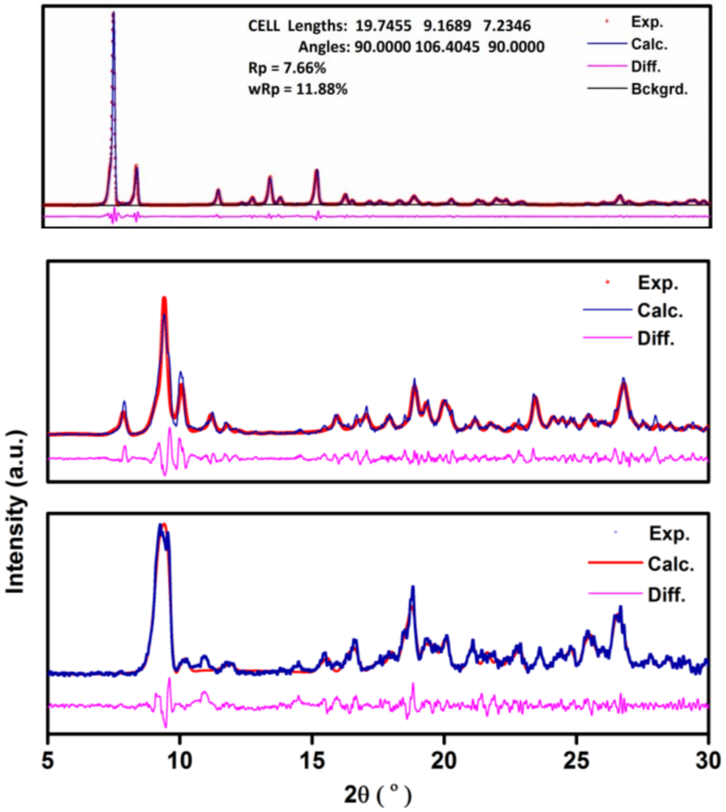

Figure s10. Pawley refinement carried out on **1** (top), **3** (middle) with a=10.400, b= 10.340, 11.292, α- 99.132°, β- 90.645°, γ- 114.645°, Rwp- 15.29, Rp- 11.30% (top) and **3_EG**, a=10.764, b= 10.605, 11.183, α- 98.274°, β- 89.992°, γ- 113.834°, Rwp- 15.23%, Rp- 11.62% (bottom).

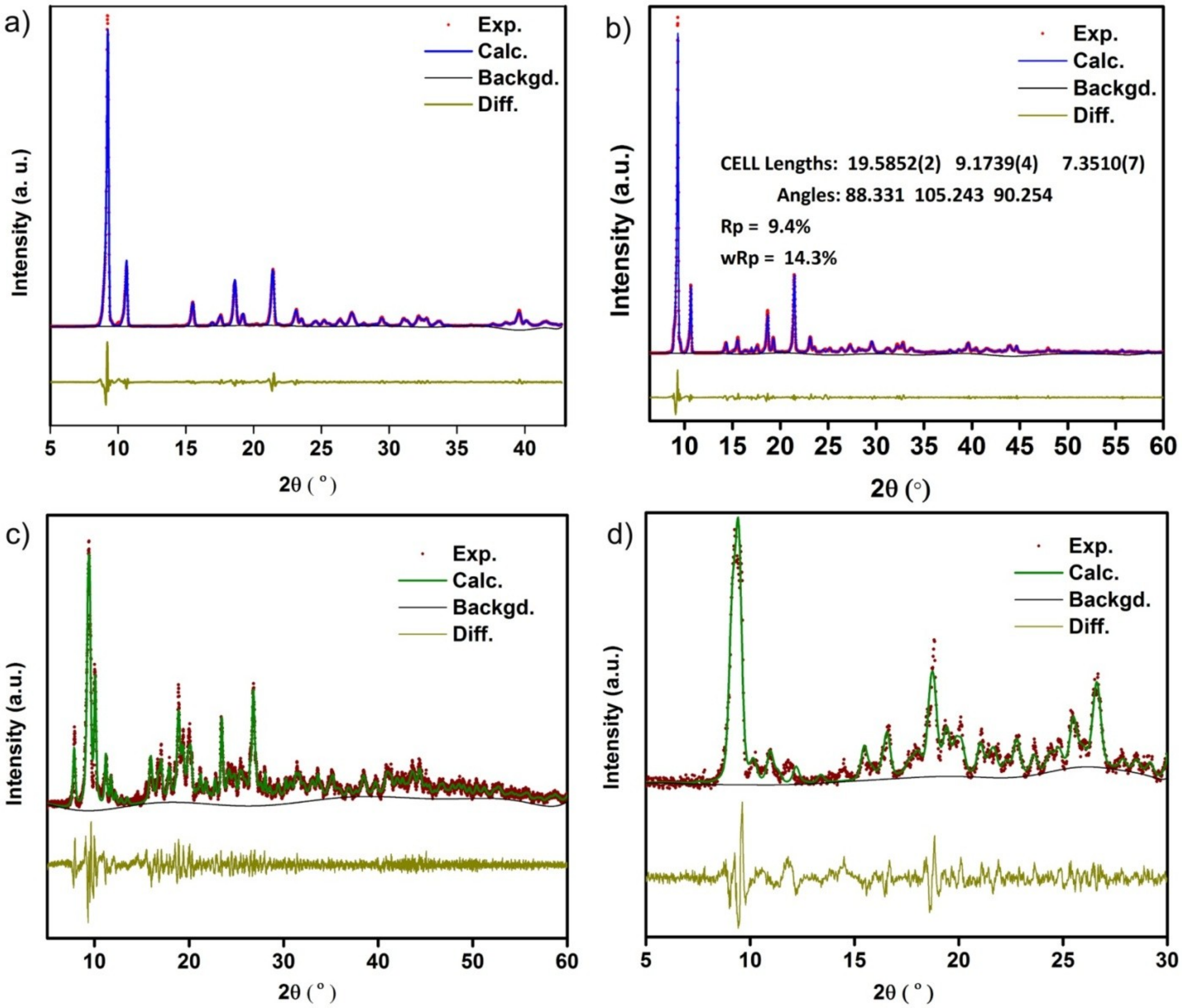

Figure s11. Le Bail fit for a) **1**, a= 19.785(2), b=9.196 (5), c=7.252 (6), α= 90.000, β=106.326 (7), γ=90.000, V= 1266.142 (19), wRp- 12.19%, Rp- 9.21%, χ2- 8.18, DWd- 0.12; b) **1_EG**, c) **3,** a= 10.322 (2), b=10.277 (2), c= 11.206 (2), α= 99.481 (12), β=90.489 (11), γ=114.909 (11), V= 1059.554 (29), wRp- 18.85%, Rp- 12.86%, χ2- 1.92, DWd- 0.45 and d**) 3_EG**, a= 10.277 (5), b=10.099 (6), c= 11.467 (6), α= 99.702 (35), β=91.683 (49), γ=113.336 (49), V= 1071.217 (123), wRp- 18.01%, Rp- 13.93%, χ2- 1.98, DWd- 0.60. *Note: Our attempts to carry out Reitveld refinements using these starting models have so far not yielded a satisfactory convergence.*

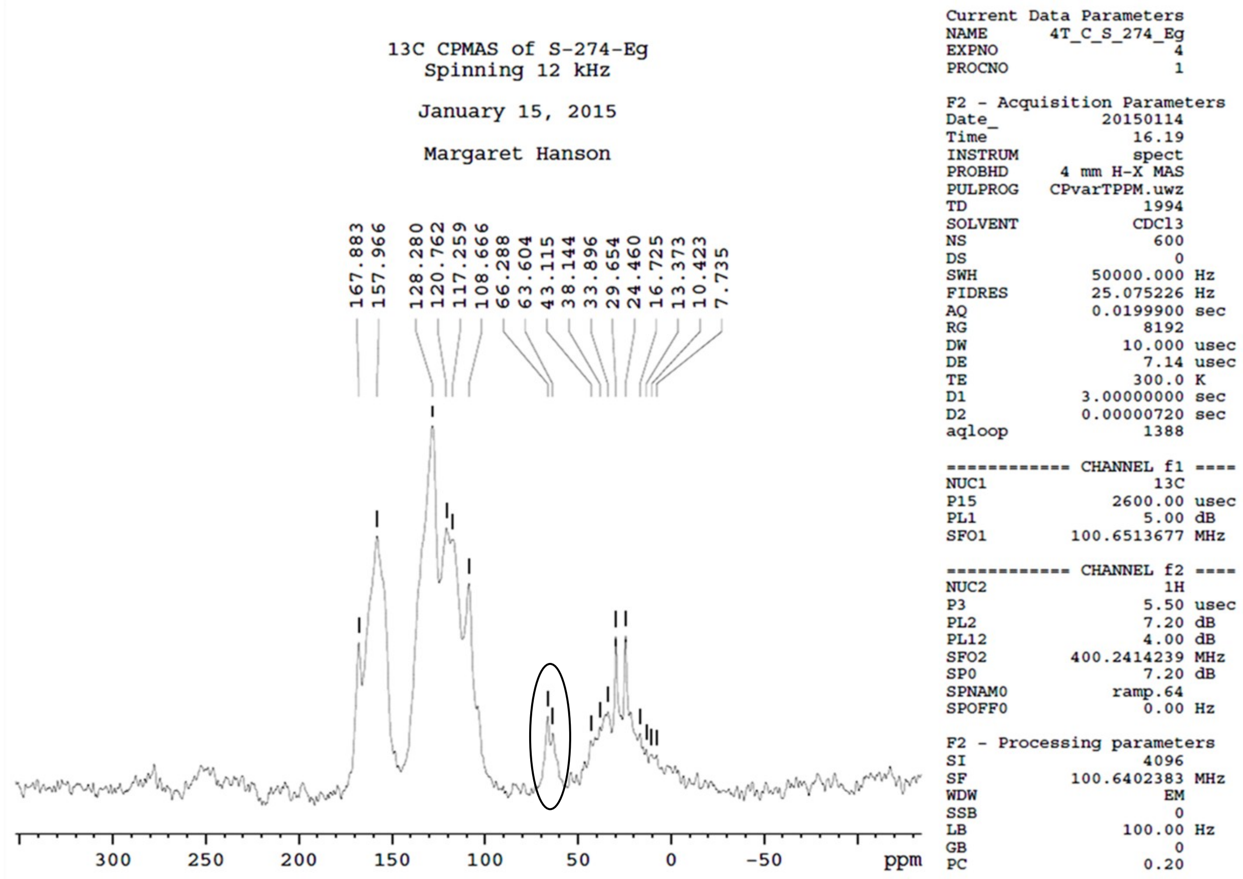

Figure s12. ^{13}C-SSNMR for **3_EG**. Peaks corresponding to EG are circled. A doublet for EG is observed which can be attributed to EG binding in a monodentate fashion to Nd through one of the OH groups.

<u>Solid State NMR Experiments for 1 and 1_EG:</u>

We used solid state NMR (SSNMR) to obtain some insights into the mobility associated with the framework components, particularly the EG and PyOH. For this purpose we have compared **1**, **1_EG** and a 'control' which is Mg-terephthalate with no pyridinol but having ethylene glycol linking two different metal centers in a μ-2 fashion via both its oxygen (**1_C**). The control represents the most rigid form of the EG and the carbons from such EG molecules should be equivalent. On the other hand the EG in **1_EG** phase has been proposed to bind to the Mg centers only via one of its oxygen, which leaves the other -OH group pendant.

^{13}C CPMAS NMR of **1**:

The ^{13}C CPMAS NMR of **1** (Figure 3c) owing to its high crystallinity showed well resolved peaks corresponding to both terephthalate and pyridinol. An important feature that was observed from this NMR was the presence of pyridinol in two forms, one wherein the pyridyl nitrogens seem to be protonated (chemical shifts: 139, 137, 121ppm) and other wherein the proton most probably resides on the -OH group bound to the metal (chemical shift: 183, 173, 129ppm). This explains the multiple peaks observed in the NMR (Figure 3c).

The region of focus is the 55-75ppm. This corresponds to peaks from EG. As can be seen from figure 3c, there is a prominent peak at 64ppm in the case of **1_EG,** which is missing in **1**. A closer look at the peak

corresponding to the EG shows that it is not a singlet, but appears to be a quartet. A simple peak fit suggests the presence of four peaks (chemical shifts: 62, 64, 65, 67ppm; χ^2= 0.99561). This indicates the presence of different coordination modes for the EG (Scheme s1). However, it does not rule out the possibility of a free EG. To gain further confidence on this coordination modes of EG, we carried out a proton decoupled ^{13}C MAS NMR of the EG loaded and it could be seen that the relative intensity of the 64ppm peak was significantly higher compared to the peak observed from the ^{13}C CPMAS NMR of the same phase (Figure 3c). This would mean the mobility associated with EG is relatively higher than those associated with the framework components.

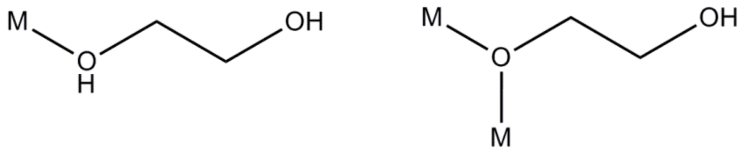

Scheme s1. Different coordination modes possible for EG binding with Mg

Now to further rule out the possibility of a free EG trapped in the MOF, we need to consider few other logics based on the inferences. Considering that the structure is quite dense, the most likely option for the EG to be accommodated would require replacement of some of the PyOH units. This EG would have to bind to Mg to maintain its coordination and prevent the collapse of the structure. Also, following the post-synthetic EG loading, the sample was subjected to aggressive washing with ethanol and methanol, to ensure that any free EG is removed.

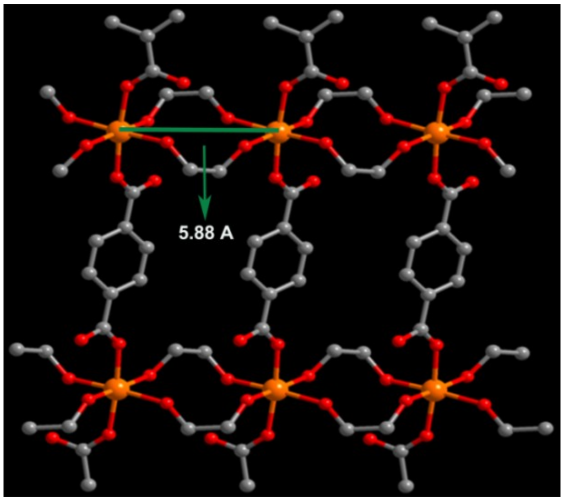

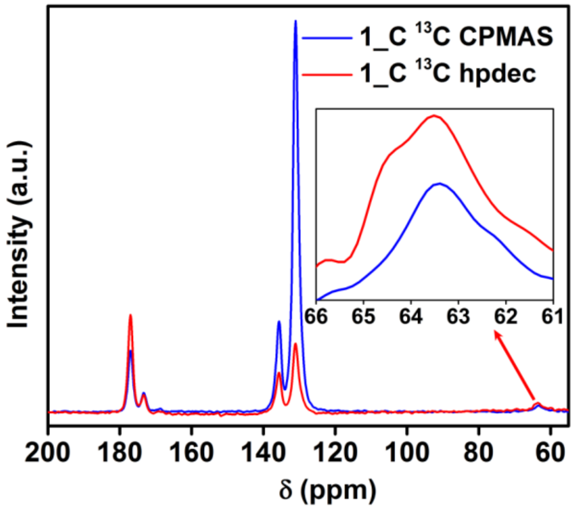

Figure s13. Left: Model compound i.e. a magnesium terephthalate ethylene glycol layer showing ethylene glycol bonded in a μ2-bridging fashion. The distance between the two magnesium centers required for accommodation of the ethylene glycol is ~5.9 Å whereas the available space in 1 is 3.6 Å. This compound has not been reported in the literature. Color code: Orange- Mg, red- O, Grey-C. Right: ^{13}C NMR for **1_C** showing the peak for EG appearing as a singlet, which suggests the presence of only one type of coordination mode for the EG molecules.

To further support this, we prepared a model compound, MgTpEG, wherein the EG are monodentately coordinated to two different Mg centers via both its -OH groups (figure s15). The ^{13}C CPMAS NMR experiments on this indicated a clear singlet for the EG peak as compared to the multiplets observed in

the case of **1-EG** (figure s16). When the ^{13}C CPMAS is compared to the ^{13}C MAS hpdec, it is clearly seen that the relative intensity and the broad line shapes corresponding to the EG are comparable, suggesting that the EG are highly bonded in this sample in comparison to the EG in **1_EG** (Figure 3c).

If the EG had been loaded as an extra-framework guest, given the extremely similar size and shape of the pores in **1** and **2** (Figure 1), both should have shown the tendency to load. However, just the Mg shows loading and there is a good chance that the EG is not loading merely as an extra-framework guest, but in addition takes up the coordination. These observations together suggest the presence of monodentately coordinating terminally bound EG in **1_EG** to be the most likely case.

Synthesis of 1_C:

This compound was synthesized hydrothermally using the approximate molar ratios $Mg(NO_3)_2{\cdot}6H_2O$, terephthalic acid and 4-hydroxypyridine (1:1). 0.1g Magnesium nitrate and 0.065 g terephthalic acid was stirred in 5 ml ethylene glycol for 15 minutes at RT. The contents were then sealed in an autoclave and heated at 140°C for 48 hours. It was slowly cooled down to room temperature. Product containing thin platy crystals was collected by filtration using methanol and acetone.

Stability Studies after proton conduction: Impedance, PXRD, SEM, TGA

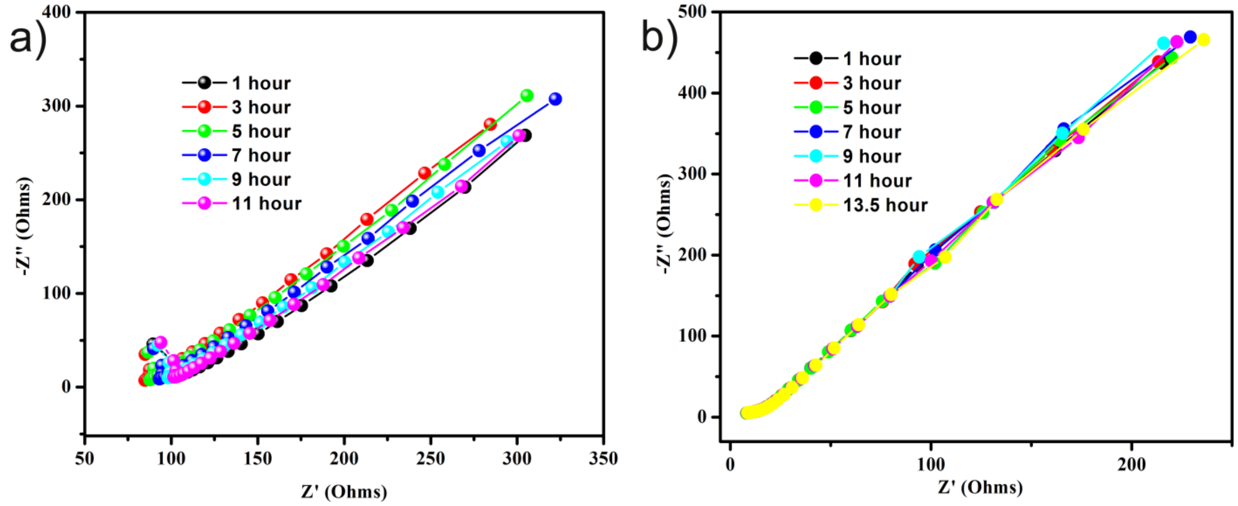

Figure s14. Impedance spectra for a) **1_EG** and b) **2** at different equilibration time at 90°C/90%RH.

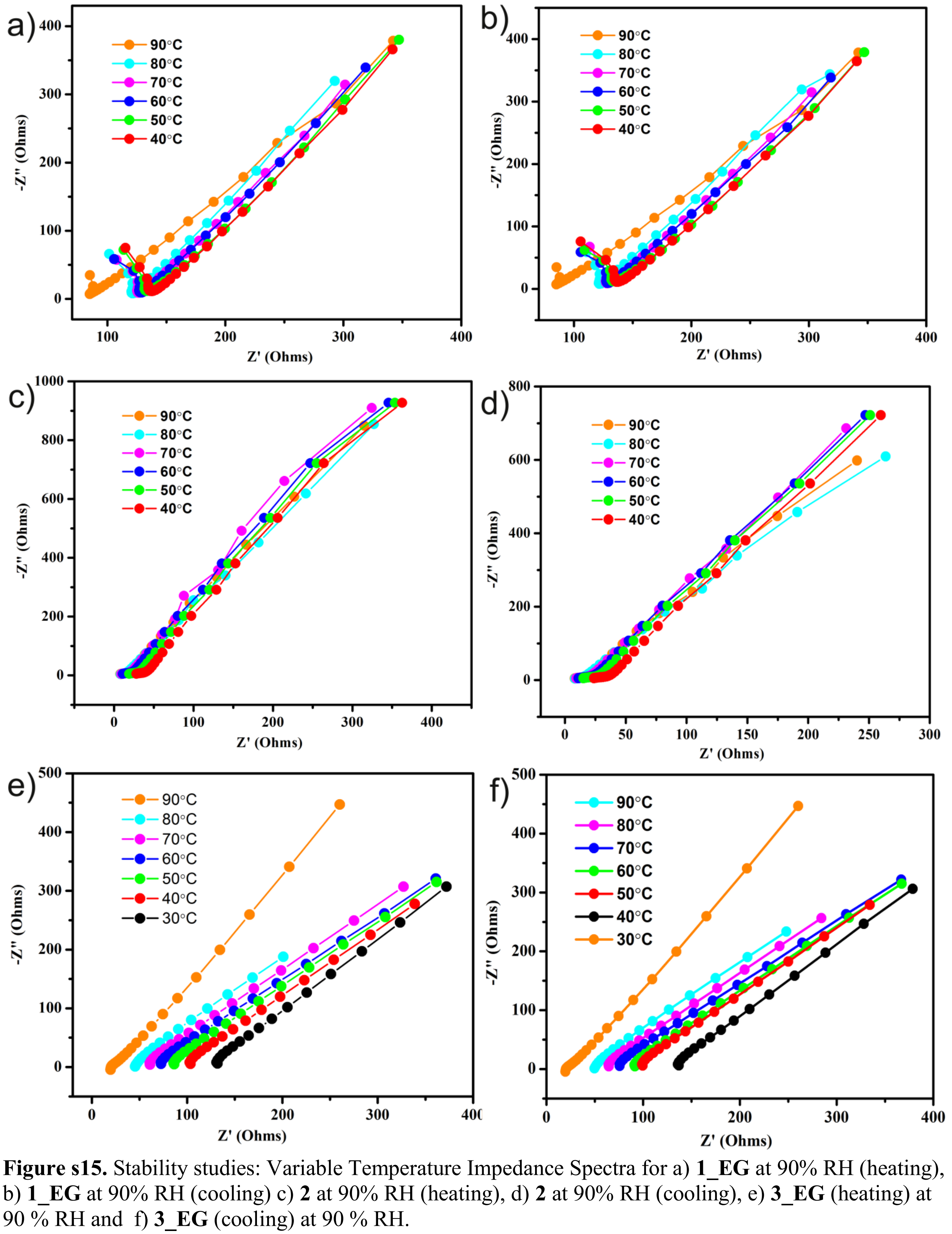

Figure s15. Stability studies: Variable Temperature Impedance Spectra for a) **1_EG** at 90% RH (heating), b) **1_EG** at 90% RH (cooling) c) **2** at 90% RH (heating), d) **2** at 90% RH (cooling), e) **3_EG** (heating) at 90 % RH and f) **3_EG** (cooling) at 90 % RH.

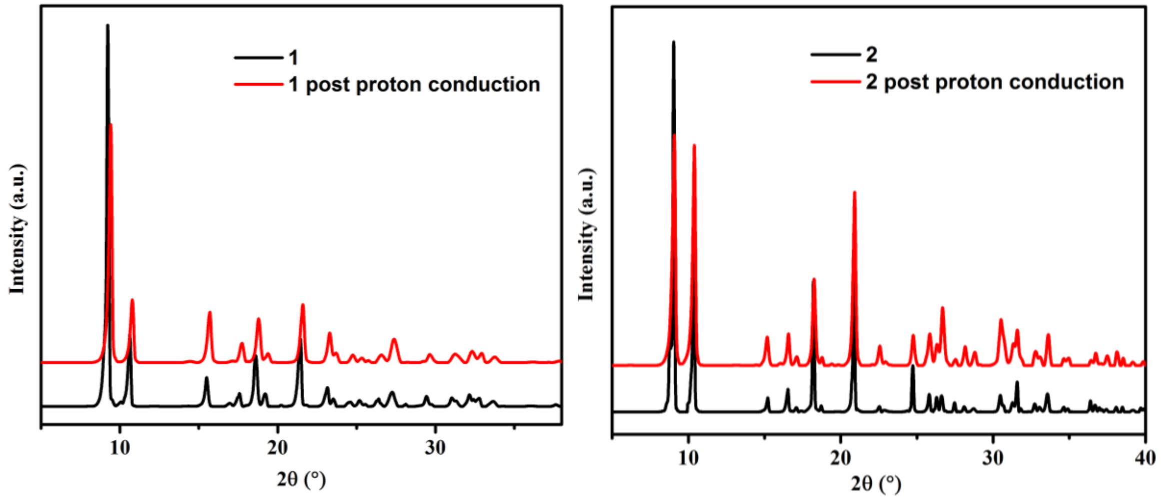

Figure s16. PXRD comparison for (left) **1** and **1** after proton conduction and (right) **2** and **2** after proton conduction.

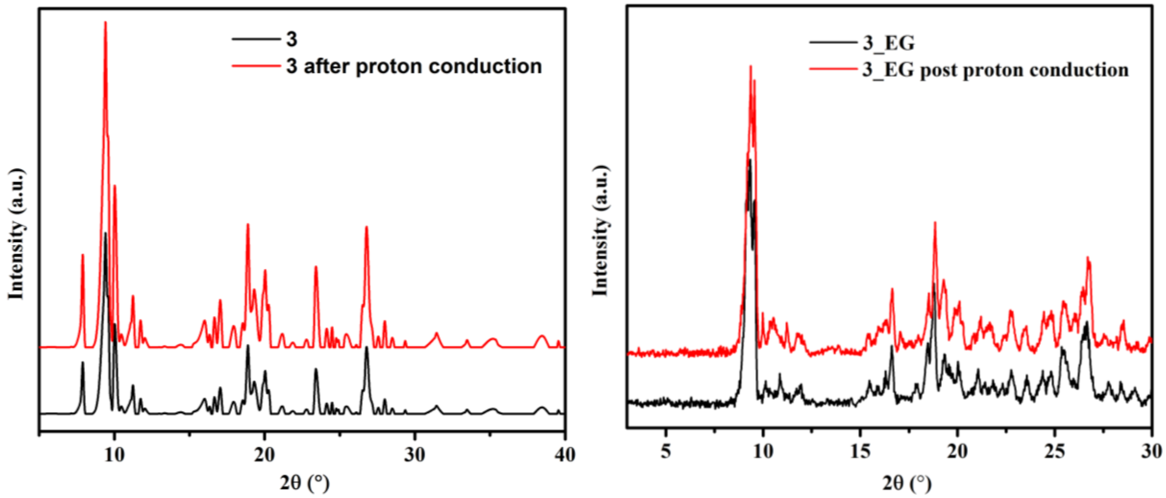

Figure s17. PXRD comparison for (left) **3** and **3** after proton conduction and (right) **3_EG** and **3_EG** after proton conduction.

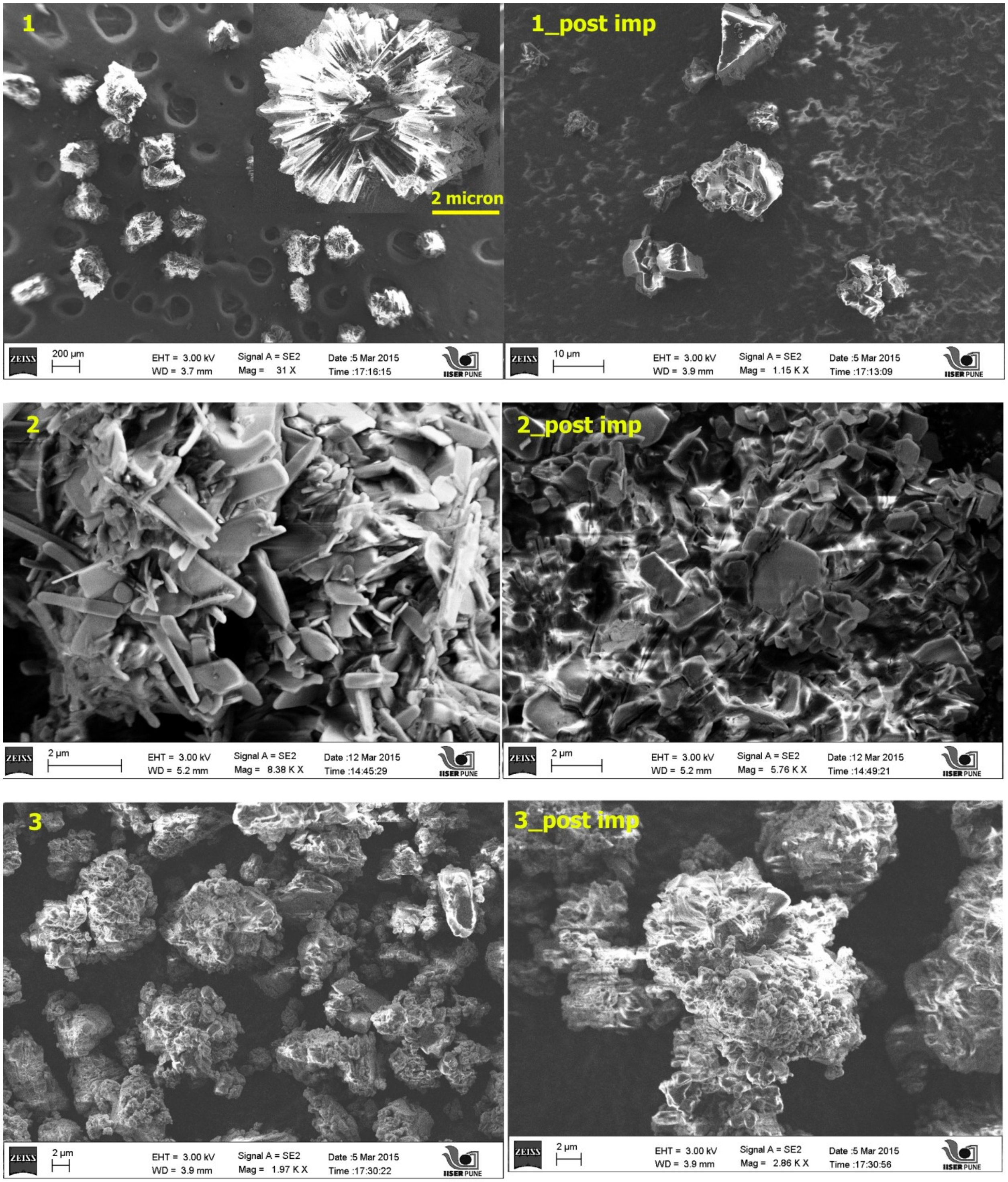

Figure s18. FE-SEM images of as-made and post-impedance samples of **1**, **2** and **3** showing the polycrystalline morphologies being intact.

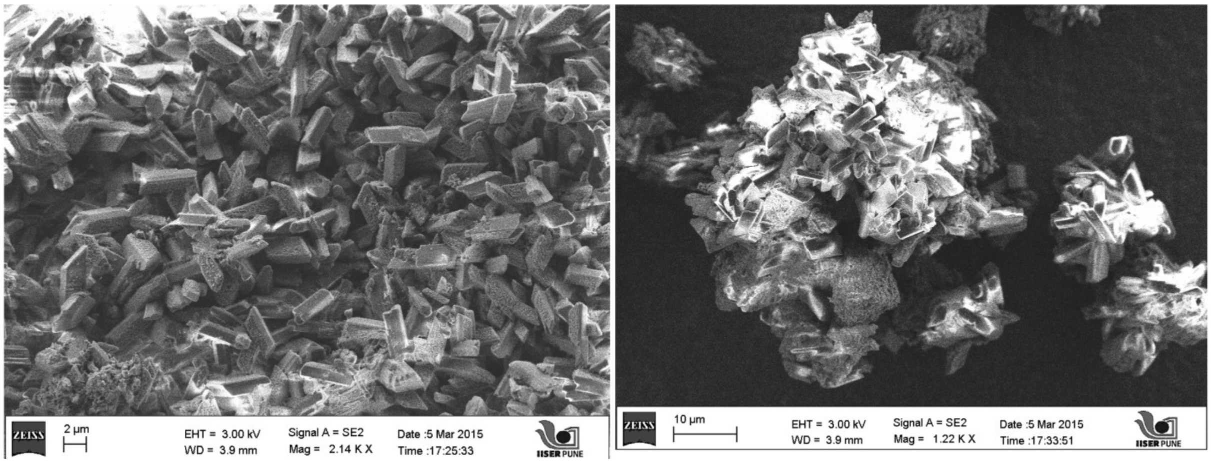

Figure s19. Field emission SEM images of the **3_EG** and the post impedance **3_EG** phases. Left: **3_EG**, Right: **3_EG** post impedance.

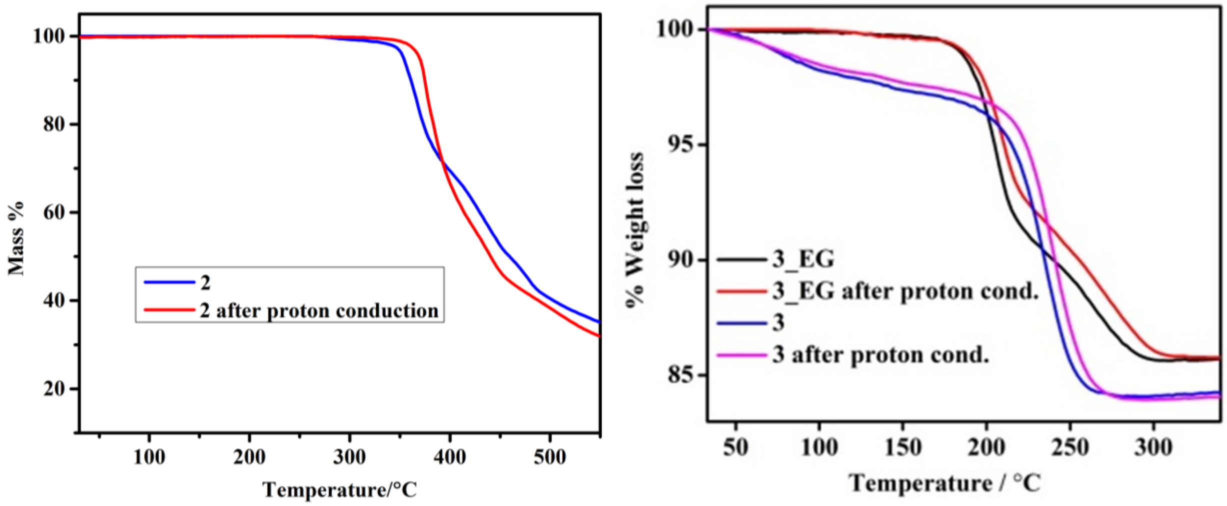

Figure s20. Comparison of the TGA plots showing good match in the observed weight losses between the as-made, EG loaded and the post treated samples, which corresponds to the loss of pyridinol.

Table s2: CHN values for all compounds before and after proton conduction.

Sample	%N	%C	%H
**1** before proton conduction	4.82	54.80	3.076
**1** after proton conduction	4.89	54.85	2.961
**1_EG** before proton conduction	3.77	52.58	3.840
**1_EG** after proton conduction	3.82	52.59	3.870
**2** before proton conduction	3.73	42.65	2.311
**2** after proton conduction	3.76	41.97	2.437
**3** before proton conduction	3.99	42.18	2.144
**3** after proton conduction	3.97	42.39	2.104
**3_EG** before proton conduction	2.69	41.82	2.191
**3_EG** after proton conduction	2.71	41.96	2.021

Appendix

Table s3. Calculation of Activation Energy for **1** @ 90% RH. Activation energies calculated using $\sigma = \sigma_0 \exp(-Ea/ [R \text{ or } (KB)^* T])$

T (°C)	R (Ω)	Thickness (cm)	A(cm^2)	κ(S/cm)	1/T (K^{-1})	Log (κ)	R(Gas constant	Slope	E_a (kJ/mol) = Slope*R
30	44185	0.0585	1.32665	9.98E-07	0.0033	-13.81752614	8.314	4080.3	33.904492
40	35356	0.0585	1.32665	1.25E-06	0.003195	-13.59460888	8.314		
50	25231	0.0585	1.32665	1.75E-06	0.003096	-13.25721417	8.314		
60	15511	0.0585	1.32665	2.84E-06	0.003003	-12.77069022	8.314		
70	11232	0.0585	1.32665	3.93E-06	0.002915	-12.44790762	8.314		
80	6973	0.0585	1.32665	6.32E-06	0.002833	-11.97118632	8.314		
90	5310	0.0585	1.32665	8.30E-06	0.002755	-11.69873261	8.314		

Table s4. Calculation of activation energy for **1_EG** (heating) at 90% RH

T (°C)	R (Ω)	Thickness (cm)	A(cm^2)	κ(S/cm)	1/T (K^{-1})	Log (κ)	R(Gas constant	Slope	E_a (kJ/mol) = Slope*R
40	138.9	0.1208	1.32665	6.55E-04	0.0033003	-7.330605999	8.314	656.3	5.4564782
50	133.5	0.1208	1.32665	6.82E-04	0.0031949	-7.290602133	8.314		
60	128.9	0.1208	1.32665	7.06E-04	0.003003	-7.255312871	8.314		
70	126.8	0.1208	1.32665	7.18E-04	0.0029155	-7.239517719	8.314		
80	120.0	0.1208	1.32665	7.59E-04	0.0028329	-7.184100982	8.314		
90	84.42	0.1208	1.32665	1.08E-03	0.0027548	-6.832080302	8.314		

Table s5. Calculation of activation energy for **1_EG** (cooling) at 90% RH

T (°C)	R (Ω)	Thickness (cm)	A(cm^2)	κ(S/cm)	1/T (K^{-1})	Log (κ)	R(Gas constant	Slope	E_a (kJ/mol) = Slope*R
90	84.42	0.1208	1.32665	1.08E-03	0.0027548	-6.832080302	8.314	633.4	5.2660876
80	121.8	0.1208	1.32665	7.47E-04	0.0028329	-7.19898467	8.314		
70	127.9	0.1208	1.32665	7.12E-04	0.0029155	-7.24752467	8.314		
60	130.0	0.1208	1.32665	7.00E-04	0.003003	-7.26450248	8.314		
50	134.4	0.1208	1.32665	6.78E-04	0.0031949	-7.297096389	8.314		
40	137.5	0.1208	1.32665	6.62E-04	0.0033003	-7.319899878	8.314		

Table s6. Calculation of activation energy for **2** (heating) at 90% RH

T (°C)	R (Ω)	Thickness (cm)	A(cm^2)	κ(S/cm)	1/T (K^{-1})	Log (κ)	R(Gas constant	Slope	E_a (kJ/mol) = Slope*R
40	56.58	0.065	1.32665	8.66E-04	0.0033003	-7.051680543	8.314	1772	14.732408
50	45.85	0.065	1.32665	1.07E-03	0.0031949	-6.841400176	8.314		
60	35.02	0.065	1.32665	1.40E-03	0.003003	-6.571944304	8.314		
70	29.52	0.065	1.32665	1.66E-03	0.0029155	-6.401092977	8.314		
80	24.52	0.065	1.32665	2.00E-03	0.0028329	-6.215514088	8.314		
90	21.02	0.065	1.32665	2.33E-03	0.0027548	-6.061499342	8.314		

Table s7. Calculation of activation energy for **2** (cooling) at 90% RH

T (°C)	R (Ω)	Thickness (cm)	A(cm^2)	κ(S/cm)	1/T (K^{-1})	Log (κ)	R(Gas constant	Slope	E_a (kJ/mol) = Slope*R
90	21.02	0.065	1.32665	2.33E-03	0.0027548	-6.061499342	8.314	1869	15.538866
80	25.31	0.065	1.32665	1.94E-03	0.0028329	-6.247224551	8.314		
70	30.02	0.065	1.32665	1.63E-03	0.0029155	-6.417888803	8.314		
60	36.32	0.065	1.32665	1.35E-03	0.003003	-6.608393531	8.314		
50	48.21	0.065	1.32665	1.02E-03	0.0031949	-6.891591445	8.314		
40	60.21	0.065	1.32665	8.14E-04	0.0033003	-7.113863428	8.314		

Table s8. Calculation of activation energy for **3_EG** (heating) at 90% RH

T (°C)	R (Ω)	Thickness (cm)	A(cm^2)	κ(S/cm)	1/T (K^{-1})	Log (κ)	R(Gas constant	Slope	E_a (kJ/mol) = Slope*R
30	131.9	0.08	1.32665	4.57E-04	0.0033003	-7.690429672	8.314	2335.93	19.419841
40	103.5	0.08	1.32665	5.83E-04	0.0031949	-7.447957225	8.314		
50	86.4	0.08	1.32665	6.98E-04	0.003096	-7.267373288	8.314		
60	72.6	0.08	1.32665	8.31E-04	0.003003	-7.093350534	8.314		
70	60.46	0.08	1.32665	9.97E-04	0.0029155	-6.910367601	8.314		
80	45.45	0.08	1.32665	1.33E-03	0.0028329	-6.624998433	8.314		
90	35.16	0.08	1.32665	1.72E-03	0.0027548	-6.368294685	8.314		

Table s9. Calculation of activation energy for **3_EG** (cooling) at 90% RH

T (°C)	R (Ω)	Thickness (cm)	A(cm^2)	κ(S/cm)	1/T (K^{-1})	Log (κ)	R(Gas constant	Slope	E_a (kJ/mol) = Slope*R
90	35.16	0.08	1.32665	1.72E-03	0.0027548	-6.368294685	8.314	2077.7	17.2739978
80	48.45	0.08	1.32665	1.24E-03	0.0028329	-6.68891795	8.314		
70	65.46	0.08	1.32665	9.21E-04	0.0029155	-6.989824881	8.314		
60	75.6	0.08	1.32665	7.98E-04	0.003003	-7.133841895	8.314		
50	90	0.08	1.32665	6.70E-04	0.0031949	-7.308195282	8.314		
40	109.2	0.08	1.32665	5.52E-04	0.0033003	-7.501566675	8.314		
30	134.5	0.08	1.32665	4.48E-04	0.0033003	-7.709949811	8.314		

5. Adsorption studies

Gas sorption isotherms were measured on Quantachrome, Autosorb iQ instrument using ultra-high purity gases (≥4.8 grade).

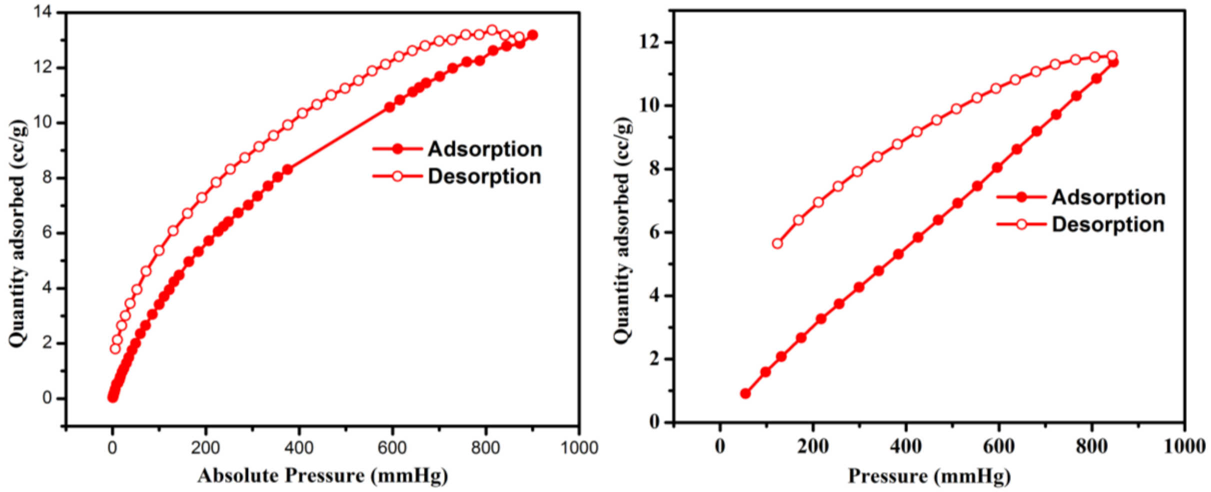

Figure s21. Left: CO_2 Adsorption isotherm for **1** at 273 K; Right: CO_2 Adsorption isotherm for **3** at 273K.
